# Comparison of Minimally Invasive Versus Abdominal Radical Hysterectomy for Early-Stage Cervical Cancer: An Updated Meta-Analysis

**DOI:** 10.3389/fonc.2021.762921

**Published:** 2022-01-24

**Authors:** Mengting Zhang, Wei Dai, Yuexiu Si, Yetan Shi, Xiangyuan Li, Ke Jiang, Jingyi Shen, Liying Ying

**Affiliations:** ^1^ The Second Clinical Medical College, Zhejiang Chinese Medical University, Hangzhou, China; ^2^ School of Basic Medical Sciences, Zhejiang Chinese Medical University, Hangzhou, China; ^3^ Department of Obstetrics and Gynecology, Ningbo Yinzhou No. 2 Hospital, Ningbo, China

**Keywords:** abdominal radical hysterectomy, early-stage cervical cancer, prognosis, meta-analysis, minimally invasive surgery

## Abstract

**Background:**

Although minimally invasive surgery (MIS) was commonly used to treat patients with early-stage cervical cancer, its efficacy remained controversial.

**Methods:**

We systematically searched PubMed, Web of Science, and Cochrane Library databases until March 2021 to compare the prognosis of early-stage cervical cancer patients who underwent MIS (laparoscopic or robot-assisted radical hysterectomy) or ARH. The primary outcomes included rates of 3- and 5-year disease-free survival (DFS) and overall survival (OS). The study protocol was registered in PROSPERO: CRD42021258116.

**Results:**

This meta-analysis included 48 studies involving 23346 patients (11220, MIS group; 12126, ARH group). The MIS group had a poorer medium-term (3-year) DFS (HR=1.08, 95% CI: 1.01-1.16, *p*=0.031) than the ARH group, without significant difference in medium-term OS as well as long-term (5-year) DFS and OS. Subgroup analysis of 3-year prognosis revealed that although patients in Western countries who underwent MIS had shorter DFS than those who underwent ARH (HR=1.10, *p*=0.024), no difference was observed in DFS among those in Asian countries. Moreover, MIS was linked to poorer 3-year DFS in patients with stage I cervical cancer (HR=1.07, *p*=0.020). Notably, subgroup analysis of 5-year prognosis revealed that patients with tumor size ≥2 cm undergoing MIS exhibited a shorter DFS than those who underwent ARH (HR=1.65, *p*=0.041).

**Conclusion:**

Patients with early-stage cervical cancer undergoing MIS may have a poorer prognosis than those undergoing ARH. Therefore, applying MIS in early-stage cervical cancer patients should be conducted with caution.

**Systematic Review Registration:**

The study protocol was registered in PROSPERO: CRD42021258116.

## Introduction

Cervical cancer was ranked as the fourth most frequently diagnosed cancer and the fourth leading cause of cancer death in women. Most of these cases occurred in sub-Saharan Africa, Melanesia, South America, and South-Eastern Asia, with the highest morbidity and mortality rates in sub-Saharan African (1). Surgery was the primary treatment option for early-stage cervical cancer to treat stage IA, IB1, and selected IIA1 cases (2). Conization alone or simple hysterectomy was an appropriate treatment option for patients with stage IA disease, whereas radical hysterectomy was the preferred treatment modality for stage IB1 or IIA1 patients (3). Abdominal radical hysterectomy (ARH) was a standard and historical treatment for early-stage cervical cancer (4, 5). As the research progressed, minimally invasive surgery (MIS) became the preferred treatment option for early-stage cervical cancer over the past two decades (6, 7).

The feasibility and safety of MIS (laparoscopic or robot-assisted radical hysterectomy) were gradually widely accepted (7, 8). Several retrospective studies and reviews (9–11) highlighted MIS benefits in reducing blood loss, shortening hospital stay, accelerating recovery time, and reducing the risk of postoperative complications, with equal survival outcomes as ARH. Nevertheless, preliminary results from a phase 3 multicenter randomized controlled trial (RCT) (12), presented at the Society of Gynecological Oncology (SGO) meeting in March 2018, indicated that early-stage cervical cancer patients undergoing MIS had a lower disease-free survival (DFS) and overall survival (OS) than those undergoing ARH. The RCT results were unexpected and sparked a huge debate (2). Since then, the guidelines from National Comprehensive Cancer Network (NCCN) (2) and International Federation of Gynecology and Obstetrics (FIGO) (13) have been revised to indicate that ARH remains the gold standard for treating early-stage cervical cancer.

Consequently, the comparison of prognosis between MIS and ARH in patients with early-stage cervical cancer remains controversial. Then, some clinical trials and reviews (14–17) demonstrated that patients with early-stage cervical cancer who underwent MIS or ARH had similar OS, but those who underwent MIS exhibited shorter DFS. Therefore, we performed a meta-analysis of available evidence to compare and evaluate medium- (3-year) and long-term (5-year) survival outcomes in patients with early-stage cervical cancer who underwent MIS or ARH.

## Methods

### Search Strategy

We conducted a systematic search of PubMed, Web of Science, and Cochrane Library to identify relevant reports published from inception until March 2021. The search terms included (uterine cervical neoplasms OR cervical cancer OR cervix cancer OR cervical carcinoma) AND (minimally invasive OR laparoscopic OR robotic OR robot-assisted OR Davinci) AND (open OR abdominal OR traditional) AND (radical hysterectomy OR surgery OR hysterectomy OR surgical procedure OR operation). Additionally, potential studies were identified by manually searching the references of included articles. From the initial search to the final selection of studies, the entire review process was mapped using the Preferred Reporting Items for Systematic Reviews and Meta-analyses (PRISMA) flow diagram. The study protocol was registered in PROSPERO: CRD42021258116.

### Eligible Criteria

The included studies met the following criteria: (1) the articles were observational studies or RCTs comparing patients with early-stage cervical cancer who underwent MIS (laparoscopic and/or robot-assisted radical hysterectomy) or ARH. (2) The studies contained detailed data on prognosis (DFS and OS) for patients with early-stage cervical cancer. (3) At least 3-year survival data was provided in the study. (4) The articles were published in English.

The exclusion criteria were as follows: (1) there was no extractable data for the study. (2) The studies included patients treated with preoperative neoadjuvant therapy or fertility-saving surgery (such as cervical resection). (3) Patients with distant metastases or those who underwent non-radical hysterectomy were investigated. (4) When a publication has been continuously updated or is duplicated, the highest-quality article is selected.

### Data Extraction and Quality Assessment

Two independent researchers extracted data from each relevant article, and a third researcher arbitrated disagreements. We recorded information on author, year of publication, country, age, number of patients, study design, MIS type, primary FIGO tumor stage, tumor size, pathologic type, lymph node metastasis, lymph-vascular space invasion, tumor differentiation, and follow-up time. Additionally, this meta-analysis used medium- and long-term prognosis endpoints, including 3- and 5-year DFS and OS. Following that, the methodological quality of included RCTs and observational studies was assessed using Cochrane Collaboration’s tool and Newcastle-Ottawa Quality Assessment Scale, respectively.

### Statistical Analysis

All statistical analyses were performed using Stata 12.0 (18). Medium- and long-term prognoses in MIS and ARH groups were analyzed using a hazard ratio (HR) with a 95% confidence interval (95% CI) (19). Moreover, heterogeneity of HR was assessed based on I^2^ statistics. Due to differences in study design and surgical treatment, a random-effects model was used to improve the credibility of results. Furthermore, Egger’s test used *p*<0.05 as the significance level to evaluate publication bias (20). Sensitivity analysis for the stability of results was performed. All statistical tests were two-sided, and *p*<0.05 was considered statistically significant.

## Results

### Characteristics of Eligible Research

**Figure 1:**
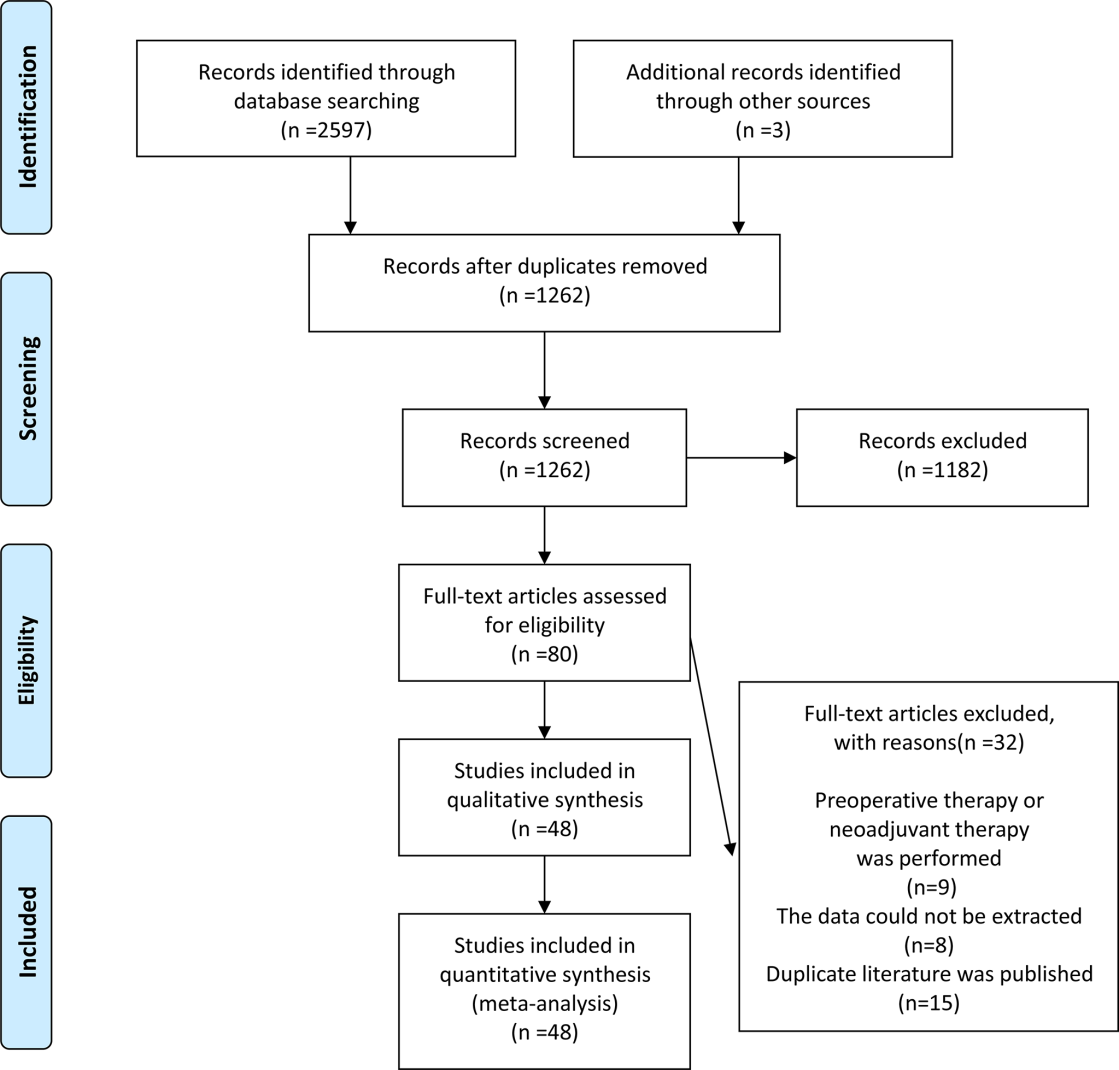
A schematic flow for the selection of articles included in this meta-analysis.

The initial search resulted in 2600 relevant studies from different electronic databases. Following screening, 48 studies (10–12, 14–17, 21–61) encompassing 23346 patients fulfilled the inclusion criteria. The detailed screening process of articles is summarized in **Figure 1**. The basic characteristics of selected studies are listed in **Table 1**. Two studies were RCTs (12, 29), and the remaining were observational studies (10, 11, 14–17, 21–28, 30–61) (n=46). Three treatment modalities were adopted by contrast: MIS (laparoscopic radical hysterectomy plus robot-assisted radical hysterectomy) versus ARH (n=10), laparoscopic radical hysterectomy versus ARH (involved 31 studies), and robot-assisted radical hysterectomy versus ARH (involved 10 studies). Of 48 studies, 23 were conducted in Asian countries and 24 in Western countries. The remaining study was conducted in both Asian and Western countries. Among the studies conducted in Asian countries, 11 were conducted in China, 10 in Korea, 1 in Singapore, and 1 in Turkey. Of the studies conducted in Western countries, 7 were conducted in America, 1 in Brazil, 1 in Canada, 1 in Denmark, 1 in France, 4 in Italy, 3 in multicenters, 2 in the Netherlands, 1 in Poland, 1 in Spain, 1 in Sweden, and 1 in the United Kingdom. All studies were published between 2004 and 2021. In these eligible studies, the number of patients was a minimum of 14 and a maximum of 1305. Almost all patients were diagnosed with FIGO stage IA-IIA cervical cancer. In addition, the proportion of patients with tumor size ≥2 cm ranged from a minimum of 18.8% to a maximum of 78.6%. Squamous cell carcinoma was the most common pathological type of cervical cancer in our study. Additional details of included studies are presented in **Supplementary Table 1**.

**Table 1:** Characteristics of all studies included in the meta-analysis.

Author	Year	Group	Country	Tumor stage (FIGO)	Patients number		Tumor size(%) ≥2cm		Pathologic type(%)							
					MIS	Control	MIS	Control	MIS				Control			
									SCC	ACC	ASC	Other	SCC	ACC	ASC	Other
Li	2021	MIS vs.ARH	Korea	IA-IIA	282	280	51.8	59.6	62.4	31.6	3.9	2.1	68.6	24.6	4.6	2.1
Kim	2021	MIS vs.ARH	Korea	IA1-IIA1	67	22	34.3	31.8	76.1	22.4	1.5	0.0	68.2	27.3	4.5	0.0
Kim	2021	MIS vs.ARH	Korea	IB1-IIA2	110	38	NA	NA	70.0	25.5	4.5	0.0	71.1	26.3	2.6	0.0
Zaccarini	2021	MIS vs.ARH	French	IA2-IIB	223	41	NA	NA	66.4	26.9	0.0	6.7	63.4	24.4	0.0	12.2
Chiva	2020	MIS vs.ARH	European	IB1	291	402	43.3	60.2	NA	NA	NA	NA	NA	NA	NA	NA
Levine	2020	MIS vs.ARH	America	IA2-IB1	82	44	42.7	54.5	43.9	47.6	8.5	0.0	65.9	27.3	6.8	0.0
Uppal	2020	MIS vs.ARH	America	IA1-IB1	560	255	18.8	31.0	55.0	40.7	4.3	0.0	60.0	35.7	4.3	0.0
Gil-Moreno	2019	MIS vs.ARH	Spain	IA1-IIB	112	76	NA	NA	60.7	33.9	0.0	5.4	61.8	30.2	0.0	7.8
Cusimano	2019	MIS vs.ARH	Canada	IA-II	473	485	NA	NA	51.6	48.4			56.1	43.9		
Ramirez^a^	2018	MIS vs.ARH	multicenter	IA1-IB1	319	312	42.3	42.9	67.10	27.30	2.80	2.80	67.30	25.60	1.90	5.10
Campos^a^	2021	LRH vs.ARH	Brazil	IA2-IIA	16	14	NA	NA	NA	NA	NA	NA	NA	NA	NA	NA
Rodriguez	2021	LRH vs.ARH	multicenter	IA1-IB1	681	698	26.5	27.8	65.0	31.1	3.9	0.0	67.0	28.1	4.9	0.0
Li	2021	LRH vs.ARH	China	IB1	574	574	NA	NA	82.4	15.2	2.4	0.0	85.2	11.5	3.3	0.0
Dai	2020	LRH vs.ARH	China	IB	213	213	NA	NA	75.6	22.5	1.9	0.0	68.5	27.2	4.2	0.0
Abel^#^	2020	LRH vs.ARH	America	II	410	1305	76.6	78.6	NA	NA	NA	NA	NA	NA	NA	NA
Kwon	2020	LRH vs.ARH	Korea	IA2-IB2	252	258	NA	NA	73.4	24.6	0.0	2.0	69.8	27.1	0.0	3.1
Qin	2020	LRH vs.ARH	China	IA2-IB1	172	84	29.7	35.7	76.8	20.3	2.9	0.0	85.7	9.5	4.8	0.0
Hu	2020	LRH vs.ARH	China	IA2-IB1/IIA1	406	406	59.1	62.1	88.0	9.1	1.7	1.2	88.9	8.4	1.5	1.2
Chen	2020	LRH vs.ARH	China	IB1	129	196	NA	NA	79.8	14.7	3.1	2.3	84.2	11.7	3.1	1.0
Wenzel	2020	LRH vs.ARH	Netherlands	IA2-IIA1	369	740	36.0	62.0	67.0	29.0	4.0	0.0	66.0	29.0	5.0	0.0
Pedone Anchora	2020	LRH vs.ARH	Italy	IA-IIB	206	217	33.5	47.5	67.5	32.5			65.0	35.0		
Wang	2019	LRH vs.ARH	China	IB2-IIB	217	179	NA	NA	86.6	11.5	1.8	0.0	89.4	5.0	5.6	0.0
Yuan	2019	LRH vs.ARH	China	IA2-IIA2	99	99	50.5	53.5	82.8	14.1	3.1	0.0	82.8	13.1	4.1	0.0
Kim	2019	LRH vs.ARH	Korea	IB	222	222	43.7	45.5	66.7	27.9	5.4	0.0	75.2	18.9	5.9	0.0
Paik	2019	LRH vs.ARH	Korea	IB1-IIA1	119	357	NA	NA	68.9	31.1	0.0	0.0	72.0	28.0	0.0	0.0
Liu	2019	LRH vs.ARH	China	IB	271	135	NA	NA	80.1	15.9	4.0	0.0	88.1	8.9	3.0	0.0
Lim	2019	LRH vs.ARH	Singapore	IA-IIA	51	85	NA	NA	41.2	49.0	3.9	5.9	58.8	31.8	3.5	5.9
Guo	2018	LRH vs.ARH	China	IA-IIA	412	139	NA	NA	82.5	17.5			79.1	20.9		
Corrado*	2018	LRH vs.ARH	Italy	IB1	152	101	NA	NA	72.3	24.3	0.0	3.4	67.3	22.8	5.9	4.0
Wang	2016	LRH vs.ARH	China	IA2-IIA2	203	203	NA	NA	84.7	11.8	3.5	0.0	76.9	18.7	4.4	0.0
Park	2016	LRH vs.ARH	Korea	IA2-IIA	196	107	52.2	27.7	NA	NA	NA	NA	NA	NA	NA	NA
Mendivil^$^	2016	LRH vs.ARH	America	IA2-IIB	49	39	NA	NA	77.6	18.4	2.0	2.0	69.2	12.8	10.3	7.7
Ditto	2015	LRH vs.ARH	Italy	IA2/IB1	60	60	NA	NA	60.0	40.0			58.0	42.0		
Toptas	2014	LRH vs.ARH	Turkey	IA2-IB1	22	46	31.8	67.4	81.8	4.6	13.6	0.0	63.0	10.9	21.7	4.4
Kong	2014	LRH vs.ARH	Korea	IB-IIA	40	48	NA	NA	75.0	17.5	7.5	0.0	81.3	14.6	4.2	0.0
van de Lande	2012	LRH vs.ARH	Netherlands	IB1	76	93	NA	NA	73.7	23.7	2.5	0.0	72.0	23.7	4.3	0.0
Choi	2012	LRH vs.ARH	Korea	IA-IIA	194	99	NA	NA	77.4	21.6	1.0	0.0	71.7	25.3	3.0	0.0
Lee	2011	LRH vs.ARH	Korea	I-II	24	48	NA	NA	79.2	16.7	4.1	0.0	79.2	16.7	4.1	0.0
Sobiczewski	2009	LRH vs.ARH	Poland	IA1-IIA	22	58	NA	NA	91.0	9.0	0.0	0.0	86.0	12.0	0.0	2.0
Malzoni	2009	LRH vs.ARH	Italy	IA1-IB1	65	62	NA	NA	86.2	10.8	3.0	0.0	85.5	9.7	4.8	0.0
Jackson	2004	LRH vs.ARH	United Kingdom	IB1	50	50	NA	NA	66.0	34.0	0.0	0.0	66.0	34.0	0.0	0.0
Abel^#^	2020	RRH vs.ARH	America	II	1234	1305	78.0	78.6	NA	NA	NA	NA	NA	NA	NA	NA
Chen	2020	RRH vs.ARH	China	IA-IIA2	879	879	NA	NA	94.1	5.2	0.7	0.0	94.3	5.2	0.0	0.0
Yang	2020	RRH vs.ARH	America	IA2-IIA	150	181	65.9	68.7	50.0	45.8	4.3	0.0	48.7	47.9	3.4	0.0
Doo	2019	RRH vs.ARH	America	IB1	49	56	39.0	62.0	61.0	33.0	6.0	0.0	57.0	36.0	7.0	0.0
Alfonzo	2019	RRH vs.ARH	Sweden	IA1-IB1	232	232	31.5	35.3	61.6	32.8	5.6	0.0	61.2	33.2	5.6	0.0
Corrado*	2018	RRH vs.ARH	Italy	IB1	88	101	NA	NA	64.8	26.1	5.8	3.3	67.3	22.8	5.9	4.0
Shah	2017	RRH vs.ARH	America	IA1-IB2	109	202	NA	NA	38.0	55.0	6.0	2.0	55.0	36.0	5.0	5.0
Sert	2016	RRH vs.ARH	Norway, America	IB1-IIA	259	232	NA	NA	57.0	36.0	3.0	4.0	59.0	35.0	3.0	3.0
Mendivil^$^	2016	RRH vs.ARH	America	IA2-IIB	58	39	NA	NA	62.1	22.4	10.3	5.2	69.2	12.8	10.3	7.7
Jensen	2020	RRH vs.ARH	Denmark	IA2-IB1	595	530	45.9	54.4	64.2	32.1	3.7	0.0	68.3	28.7	3.0	0.0

MIS, Minimally invasive surgery; ARH, Abominal radical hysterectomy; LRH, Laparoscopic radical hysterectomy; RRH, Robot-assisted radical hysterectomy; FIGO, International Federation of Gynecology and Obstetrics; RCT, Randomized controlled trial; POBS, Prospective observational study; ROBSs, Retrospective observational studies; SCC, Squamous cell carcinoma; ACC, Adenocarcinoma; ASC, Adenosquamous carcinoma; NA, Not available.

a These two studies were RCTs, and the rest were observational studies (including 1 POBS and 45 ROBSs.).

^#^Both were from the same study.

*Both were from the same study.

^$^Both were from the same study.

### Quality Assessment

Cochrane collaboration’s tool was employed to assess the quality of RCT enrolled in the study. For observational studies, quality assessment was performed based on the Newcastle-Ottawa Quality Assessment Scale, and only high-quality studies with a total score ≥6 were included in the final analysis. Details on quality assessment are displayed in **Supplementary Table 2**.

### Prognostic Analysis

**Figure 2:**
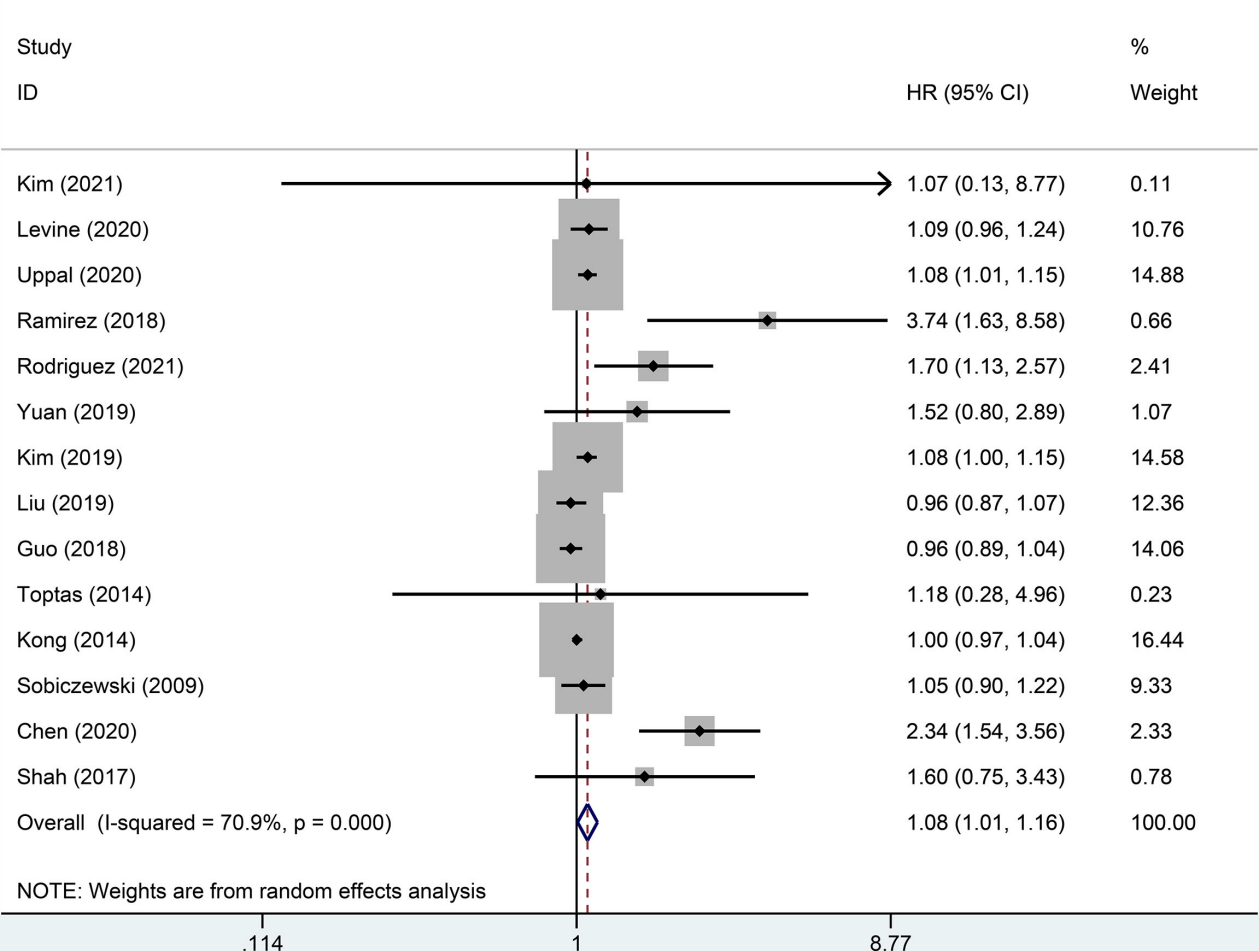
Forest plot of the 3-year disease-free survival (DFS) of patients with early-stage cervical cancer on minimally invasive surgery (MIS) (p=0.031).

**Figure 3:**
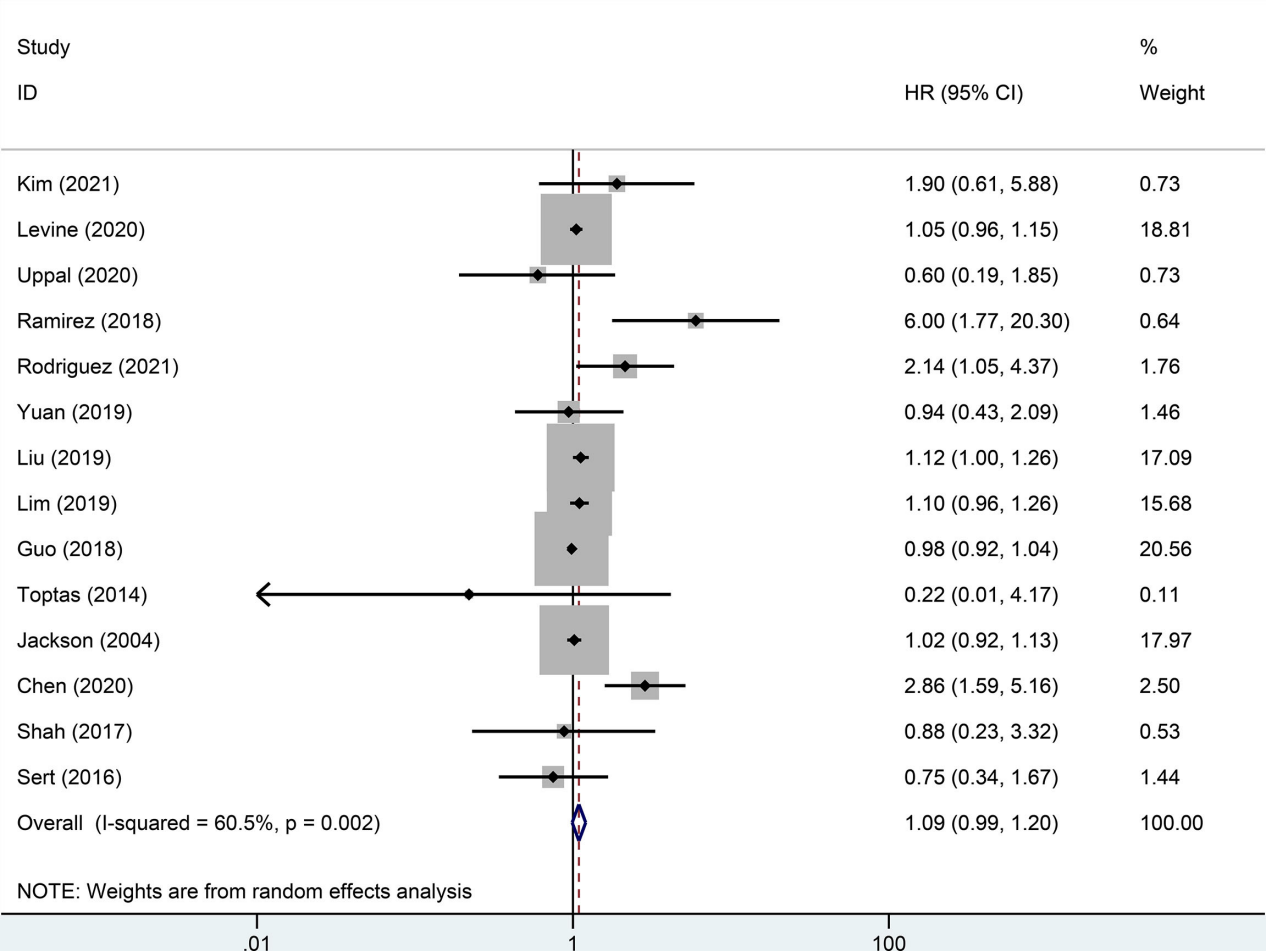
Forest plot of the 3-year overall survival (OS) of early-stage cervical cancer patients on minimally invasive surgery (MIS) (p=0.082).

Random effect analysis of 14 of 48 reports on 3-year DFS encompassing 7003 patients with early-stage cervical cancer revealed a statistically significant difference, suggesting that the MIS group had a shorter 3-year DFS than the ARH group (HR=1.08, 95% CI: 1.01-1.16, *p*=0.031; **Figure 2**). Besides, 14 studies involving 7118 patients with early-stage cervical cancer were assessed for 3-year OS, without observing a significant difference in 3-year OS between MIS and ARH groups (HR=1.09, 95% CI: 0.99-1.20, *p*=0.082; **Figure 3**).

**Figure 4:**
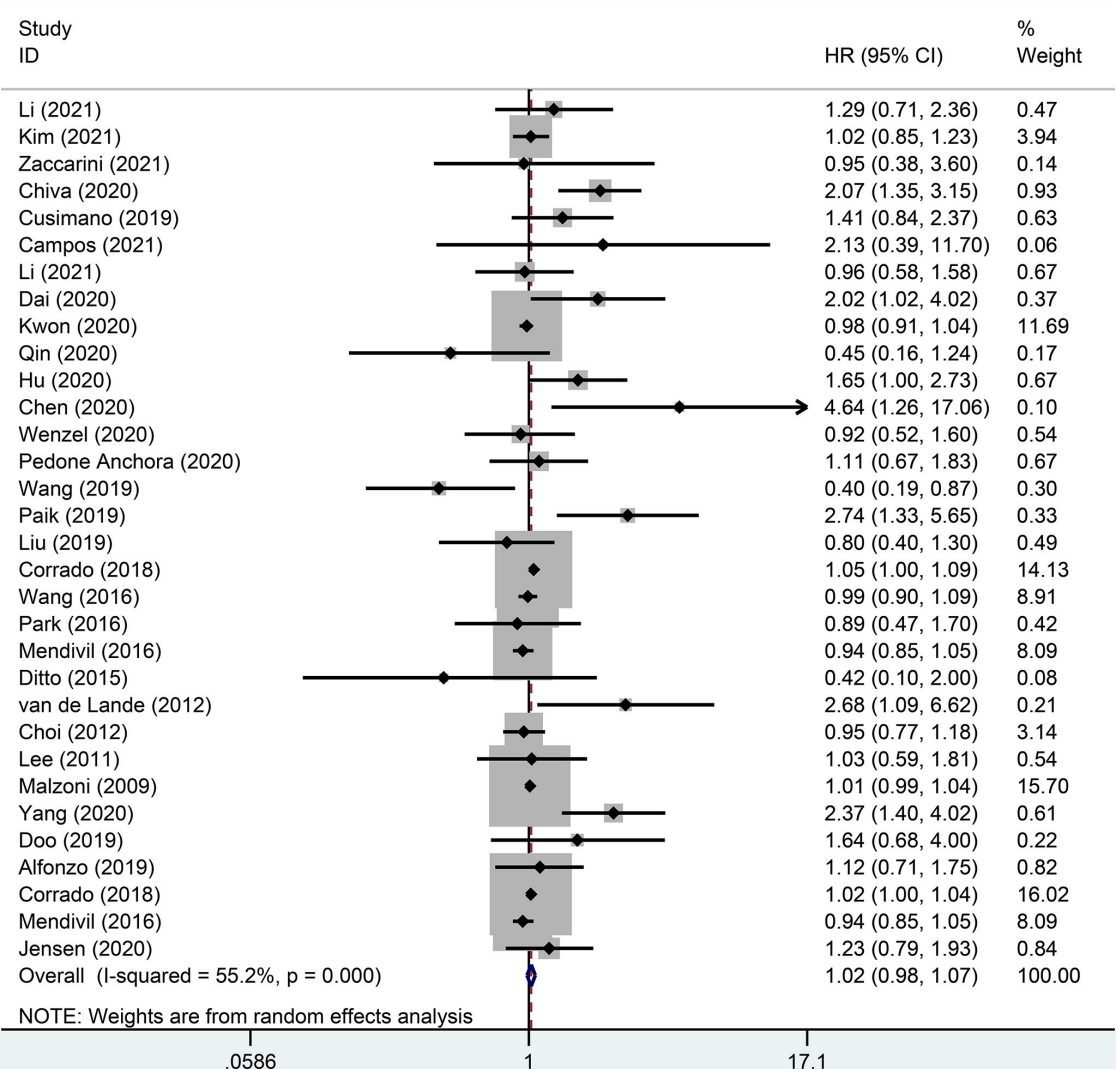
Forest plot for the 5-year disease-free survival (DFS) of early-stage cervical cancer patients on minimally invasive surgery (MIS) (p=0.266).

**Figure 5:**
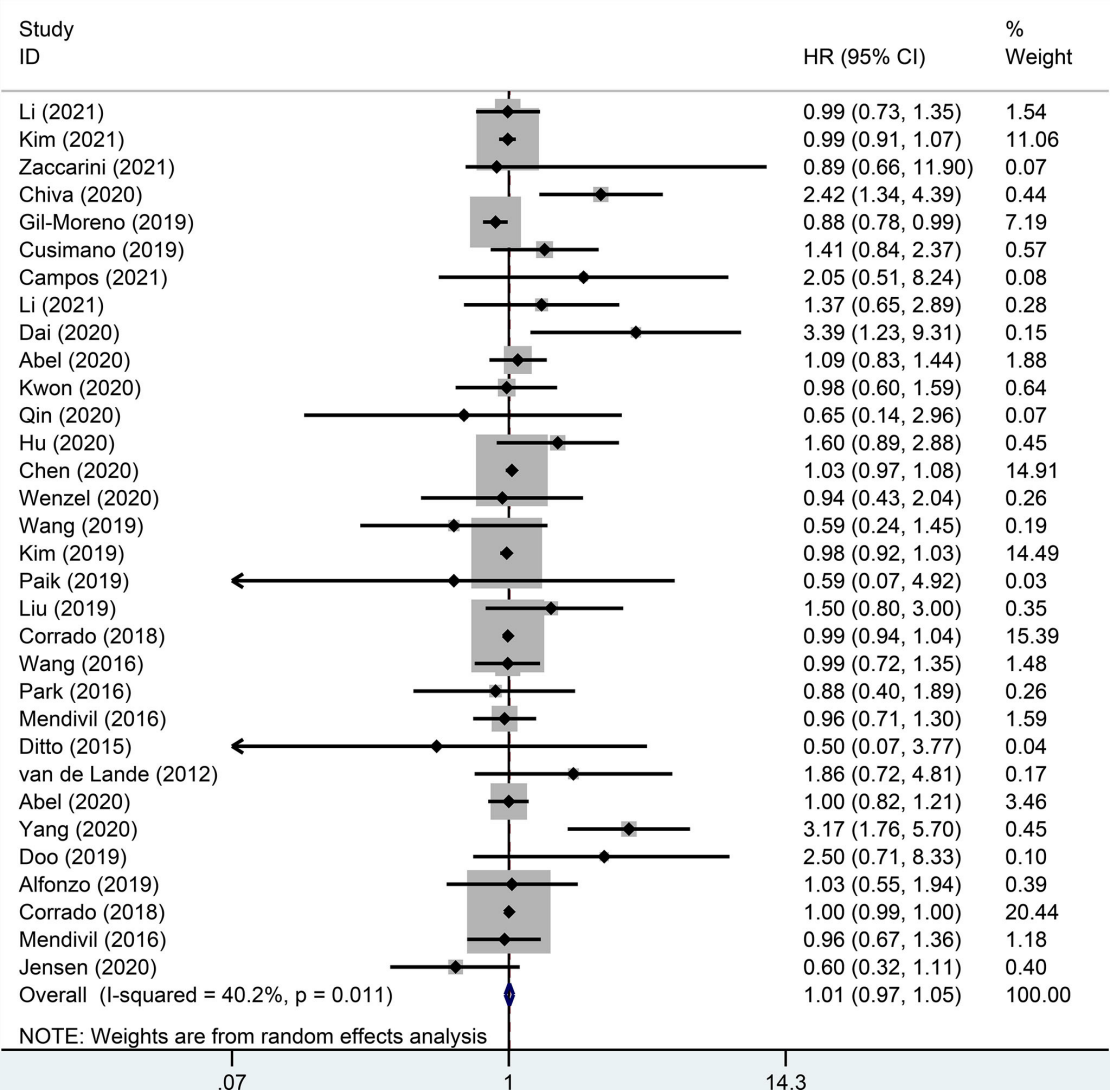
Forest plot for the 5-year overall survival (OS) of early-stage cervical cancer patients on minimally invasive surgery (MIS) (p=0.795).

There were 32 studies that provided 5-year DFS, including 13025 patients with early-stage cervical cancer. Among them, there were 6471 cases in the MIS group and 6312 cases in the ARH group. The results revealed that MIS had no significant effect on long-term DFS in patients with early-stage cervical cancer compared with ARH (HR=1.02, 95% CI: 0.98-1.07, *p*=0.266; **Figure 4**). Moreover, 15551 patients were evaluated for 5-year OS in 32 studies. The results illustrated that long-term OS of patients undergoing MIS was similar to that of patients undergoing ARH (HR=1.01, 95% CI: 0.97-1.05, *p*=0.795; **Figure 5**).

### Subgroup Analysis of 3- and 5-Year Survival

Of 14 studies that provided 3-year DFS, a subgroup analysis was performed on cervical cancer stage, including nine studies for stage I (HR=1.07, 95% CI: 1.01-1.14, *p*=0.020) and five studies for IA-IB1 (HR=1.23, 95% CI: 1.02-1.49, *p*=0.034). Patients with stages I and IA-IB1 who underwent MIS had a poorer 3-year DFS than those with ARH. Additionally, five studies were conducted in Western countries (HR=1.10, 95% CI: 1.01-1.20, *p*=0.024), whereas eight studies were conducted in Asian countries (HR=1.04, 95% CI: 0.96-1.12, *p*=0.381). Overall, the pooled subgroup results demonstrated that in Western countries, patients treated with MIS exhibited a significantly shorter 3-year DFS than those treated with ARH. Nevertheless, in studies of Asian countries, no difference in DFS was observed between MIS and ARH groups. Regarding patients with tumor size <2 cm, a subgroup analysis revealed no statistically significant difference in 3-year DFS between both groups (HR=1.04, 95% CI: 0.98-1.09, *p*=0.186) (**Table 2**).

**Table 2:** Subgroup analysis of the 3- and 5-year survival of early-stage cervical cancer patients.

	No. of studies	HR	95%CI	*p*	Heterogeneity (I^2^) (%)
3-year disease-free survival					
I	9	1.07	1.01-1.14	*0.020*	59.6
IA-IB1	5	1.23	1.02-1.49	*0.034*	69.2
Western	5	1.10	1.01-1.20	*0.024*	30.9
Asia	8	1.04	0.96-1.12	0.381	69.3
Tumor size<2cm	3	1.04	0.98-1.09	0.186	0.0
3-year overall survival					
I	9	1.06	0.99-1.14	0.096	48.4
IA-IB1	5	1.42	0.71-2.85	0.321	70.4
IB1-II	3	1.02	0.92-1.13	0.689	0.0
Western	6	1.04	0.95-1.14	0.360	13.0
Asia	7	1.12	0.96-1.31	0.134	69.4
Tumor size<2cm	3	1.01	0.98-1.05	0.441	17.4
Tumor size≥2cm	2	2.26	0.64-7.94	0.203	71.1
5-year disease-free survival					
I	16	1.03	0.98-1.09	0.247	68.5
IA-IB1	13	1.04	0.99-1.09	0.157	65.0
IB1-II	12	1.06	0.99-1.13	0.111	73.2
Western	17	1.03	0.98-1.07	0.243	58.3
Asia	15	1.03	0.92-1.17	0.577	54.4
Tumor size<2cm	9	0.86	0.54-1.37	0.530	50.9
Tumor size≥2cm	6	1.65	1.02-2.66	*0.041*	69.6
5-year overall survival					
I	16	1.01	0.96-1.06	0.665	56.2
IA-IB1	13	1.01	0.97-1.05	0.626	37.2
IB1-II	14	1.01	0.97-1.06	0.606	51.4
II	2	1.03	0.88-1.20	0.745	0.0
Western	18	1.02	0.95-1.10	0.597	55.1
Asia	14	1.00	0.96-1.04	0.900	6.3
Tumor size<2cm	9	1.03	0.98-1.07	0.250	0.0
Tumor size≥2cm	5	1.76	0.97-3.19	0.063	65.1

Likewise, there were 14 studies that reported 3-year OS, with nine reporting stage I (HR=1.06, 95% CI: 0.99-1.14, *p*=0.096), five reporting stage IA-IB1 (HR=1.42, 95% CI: 0.71-2.85, *p*=0.321) and three reporting stage IB1-II (HR=1.02, 95% CI: 0.92-1.13, *p*=0.689). The subgroup analysis indicated that compared with patients undergoing ARH, no statistical difference was observed in 3-year OS in patients with stage I, IA-IB1, and IB1-II cervical cancer undergoing MIS. In addition, six studies were conducted in Western countries (HR=1.04, 95% CI: 0.95-1.14, *p*=0.36), while the remaining seven were conducted in Asian countries (HR=1.12, 95% CI: 0.96-1.31, *p*=0.134). Additionally, a subgroup analysis based on tumor size revealed that compared with patients undergoing ARH, those with early-stage cervical cancer undergoing MIS included tumor size <2 cm (HR=1.01, 95% CI: 0.98-1.05, *p*=0.441) and tumor size ≥2 cm (HR=2.26, 95% CI: 0.64-7.94, *p*=0.203), but no statistically significant difference was observed in 3-year OS (**Table 2**).

Of 32 studies on 5-year DFS, 16 were for stage I cervical cancer (HR=1.03, 95% CI: 0.98-1.09, *p*=0.247), 13 were for stage IA-IB1 (HR=1.04, 95% CI: 0.99-1.09, *p*=0.157), and 12 were for stage IB1-II (HR=1.06, 95% CI: 0.99-1.13, *p*=0.111). A total of 17 of 32 studies were conducted in Western countries (HR=1.03, 95% CI: 0.98-1.07, *p*=0.243), whereas 15 studies were conducted in Asian countries (HR=1.03, 95% CI: 0.92-1.17, *p*=0.577). Furthermore, nine studies evaluated 5-year DFS in patients with tumor size <2 cm (HR=0.86, 95% CI: 0.54-1.37, *p*=0.53). All above subgroup analyses revealed that MIS was not linked to long-term DFS in patients with early-stage cervical cancer. Notably, for early-stage cervical cancer patients with tumor size ≥2 cm, the pooled results disclosed that the MIS group may have a significantly shorter 5-year DFS than ARH group (HR=1.65, 95% CI: 1.02-2.66, *p*=0.041) (**Table 2**).

5-year OS was assessed in 32 studies, including 16 for stage I cervical cancer (HR=1.01, 95%CI: 0.96-1.06, *p*=0.665), 13 for stage IA-IB1 (HR=1.01, 95% CI: 0.97-1.05, *p*=0.626), 14 for stage IB1-II (HR=1.01, 95% CI: 0.97-1.06, *p*=0.606), and 2 for stage II (HR=1.03, 95% CI: 0.88-1.20, *p*=0.745). Moreover, 18 of 32 studies were performed in Western countries (HR=1.02, 95% CI: 0.95-1.10, *p*=0.597), while the remaining studies (n=14) were conducted in Asian countries (HR=1.00, 95% CI: 0.96-1.04, *p*=0.9). Briefly, in the above subgroup analyses, the results revealed that MIS was not correlated with 5-year OS in patients with early-stage cervical cancer. Additionally, subgroup analyses of 5-year OS were performed for patients with various tumor sizes. According to the results for patients with tumor size <2 cm, long-term OS was not statistically different between the MIS and ARH groups (HR=1.03, 95% CI: 0.98-1.07, *p*=0.25). Although patients with tumor size ≥2 cm undergoing MIS had a poorer long-term OS than those undergoing ARH, the difference was not statistically significant (HR=1.76, 95% CI: 0.97-3.19, *p*=0.063) (**Table 2**).

### Publication Bias and Sensitivity Analysis of 3- and 5-Year Survival

Egger’s test revealed no significant publication bias in this meta-analysis (*p*>0.05). Additionally, sensitivity analysis of 3- and 5-year survival rates revealed that the results remained stable.

## Discussion

Academics had cast doubt on previous surgical findings following the publication of 2018 Laparoscopic Approach to Cervical Cancer (LACC) trial. Additionally, the impact of MIS and ARH on the prognosis of patients with early-stage cervical cancer has been controversial. The RCT findings (12) revealed that MIS was associated with poorer DFS and OS than ARH, but some limitations were found. On the one hand, the trial of patients in the MIS group was terminated prematurely, and some patients received insufficient follow-up time. On the other hand, the results were inapplicable to assessing survival outcomes in “low-risk” cervical cancer patients. Moreover, the trial lacked specific preoperative imaging and central pathology (62). As a result, this meta-analysis systematically evaluated and compared prognosis (3- and 5-year DFS and OS) of patients with early-stage cervical cancer in MIS and ARH groups, and subgroup analyses of associated factors were conducted.

Our meta-analysis included 48 studies with 23346 patients. Based on evaluating 3-year prognosis, the results revealed that patients with early-stage cervical cancer undergoing MIS had a poorer 3-year DFS than those undergoing ARH, without observing a statistical difference in 3-year OS. In a multi-institution retrospective study, Uppal et al. (15) indicated that patients undergoing MIS had a poorer DFS, but no difference was observed in OS compared to those undergoing ARH, consistent with our findings. Meanwhile, subgroup analyses of tumor stage, region, and tumor size were performed on a 3-year prognosis. The pooled results of 3-year prognosis revealed that patients with stage I cervical cancer undergoing MIS exhibited poorer DFS than those undergoing ARH. Similarly, the pooled results in 3-year DFS demonstrated that patients with stage IA-IB1 and Western countries undergoing MIS indicated a shorter DFS than those undergoing ARH. Besides, no significant difference was observed in other subgroup analyses. For patients with stage I and IA-IB1, the poor results may be influenced by the frequency of the use of postoperative adjuvant therapy. In a Norwegian study (63), the incidence of postoperative radiotherapy was low in the MIS and ARH groups (6.1% vs. 12.5%). This study indicated that early-stage cervical cancer patients with stage IB1 and tumor size ≤2 cm who underwent MIS had significantly worse DFS than those with ARH. Nevertheless, a population-based study from Denmark (FIGO Stage IA2-IB1) (61) and a population-based study from Sweden (FIGO Stage IA1-IB1) (58) indicated a relatively high incidence of adjuvant therapy in the MIS and ARH groups (21.9% vs. 31.9%; 30.6% vs. 31.9%, respectively), with no difference in survival outcomes between the two groups. Since there was no difference in surgical techniques among the 3 closely related countries, it was tempting to speculate that the use of adjuvant radiotherapy had an impact on the survival outcomes. This speculation needs to be further confirmed. For patients from Western countries, DFS of MIS group was obviously inferior to that of ARH group. There was no clear explanation for this result. We suspected that it may be related to the different types of adjuvant therapy available in different geographic areas. Additionally, the frequency of use of adjuvant treatment in a study may influence the result. The relatively high number of patients receiving adjuvant therapy was likely to reduce the difference in survival between the two groups. NCCN guidelines (2) stated that for patients with stage IA2, IB1, or IIA1 who had negative lymph nodes after surgery but had other risk factors, pelvic external-beam radiation therapy was recommended with (or without) concurrent chemotherapy. A multicenter retrospective study from some Western countries (30) implicated that the incidence of postoperative adjuvant therapy was lower in the MIS group and the ARH group (29.2% vs. 30.7%), and the MIS group had worse prognosis after adjuvant therapy adjustment. By contrast, in a multi-center retrospective study from China (31), postoperative adjuvant therapy consisted of chemotherapy, radiotherapy or chemoradiotherapy. The study implicated that the incidence of postoperative adjuvant therapy was relatively high in the MIS and ARH groups (57.7% vs. 59.6%), with no significant difference between the two groups. Unfortunately, additional confirmation is required due to the scarcity of studies on adjuvant therapy and prognosis.

Likewise, 5-year prognosis was assessed in patients with early-stage cervical cancer. The results demonstrated no statistically significant difference between MIS and ARH groups. Brandt et al. (43) evaluated 196 cases and presented similar results that MIS had no association with a 5-year prognosis in patients with early-stage cervical cancer. Additionally, Abel et al. (33) revealed that stage II cervical cancer undergoing MIS or ARH revealed comparable 5-year survival rates. Nonetheless, some recent studies have demonstrated that early-stage patients undergoing MIS had poorer DFS and OS than those undergoing ARH. According to Dai et al. (32), patients with stage IB undergoing ARH had better DFS and OS than those undergoing MIS (5-year DFS rate, 94.1% vs. 87.5%; 5-year OS rate, 98.1% vs. 92.3%). Chiva et al. (25) reported in an international European cohort observational study that early-stage cervical cancer patients undergoing MIS increased the risk of recurrence and death compared with those undergoing ARH. In addition, we observed that in many retrospective studies (28, 30, 33, 41, 47, 51, 57, 59), the MIS group had a shorter follow-up time than that of the ARH group. In order to objectively evaluate the effect of MIS and ARH on the prognosis of patients with early-stage cervical cancer, more adequate follow-up time is needed. Furthermore, 5-year prognosis subgroups were analyzed based on tumor stage, region, and tumor size. Except for tumor size ≥2 cm, no statistical difference was observed in other subgroup analyses. The lack of discrepancy in stage II may be due to the relatively small number of studies.

Specifically, the pooled results revealed that patients with tumor size ≥2 cm treated with MIS had a poorer long-term prognosis than those treated with ARH. Consistent with the results of Li et al. (21) and Chen et al. (64), patients with tumor size ≥2 cm undergoing MIS had a shorter DFS than those treated with ARH. The following reasons may be responsible for poorer DFS in patients with tumor size ≥2 cm undergoing MIS: (1) Wagner et al. (65) pointed out that tumor size was an independent prognostic factor for each stage and greatly influenced the prognosis of cervical cancer patients. Larger tumors have a higher risk of lymphatic metastasis (66–69), requiring greater tumor resection (66). However, MIS might be less thoroughly resected than ARH. (2) Pressing the tumor while using a uterine manipulator may spread cancer or increase lymphatic vascular space infiltration (70–72). The SUCCOR study (25) indicated that the risk of recurrence was 2.76-fold higher in patients undergoing MIS with a uterine manipulator compared to those undergoing ARH. (3) When tumors are large, selection bias of surgical methods may affect the results (73). MIS probably brings some surgical difficulties to surgeons (21, 22, 66, 70, 74), reducing the surgical effect. (4) Pneumoperitoneum environment may be a prognostic factor in patients undergoing MIS. An *in vitro* study (75) demonstrated that when cervical cancer cells were stimulated in CO_2_ pneumoperitoneum environment *in vitro*, their proliferation ability was enhanced following a short period of inhibition. A retrospective analysis by Kong et al. (76) found that patients with early-stage cervical cancer undergoing MIS in pneumoperitoneal conditions increased the risk of recurrence and intraperitoneal tumor spread. In addition, the SUCCOR study (25) proposed that implementing a preoperative protective vaginal closure in patients undergoing MIS dramatically reduced the risk of recurrence and peritoneal metastasis compared to those undergoing ARH.

Compared with other studies, the strengths of this meta-analysis included the division of patients’ prognoses into medium- (3-year) and long-term (5-year) categories, as well as subgroup analyses for various factors such as tumor stage, region, and tumor size. Indeed, our meta-analysis had several limitations. First, only two of the included studies were RCTs, while the remaining were observational studies, resulting in inevitable risks such as selection bias. Second, the baseline characteristics of studies varied, such as tumor stage and surgical procedure. Besides, sentinel lymph node and adjuvant therapy assessments were not performed due to limited data. Furthermore, the sample size of our study was impacted by language restrictions associated with included literature. Finally, the retrieval time span was relatively long, allowing for MIS technology development, resulting in studies that may not accurately reflect changes in survival outcomes over time.

## Conclusion

In patients with Western countries and stage I cervical cancer, MIS was linked to a shorter medium-term DFS, particularly in stage IA-IB1. Regarding long-term prognosis, patients with tumor size ≥2 cm were unsuitable for MIS and had shorter DFS than ARH. Accordingly, MIS should be chosen with caution in patients with early-stage cervical cancer. Nevertheless, more large-scale RCTs, including two ongoing trials (NCT03739944, NCT03719547), and clinical studies are required to provide relevant data.

## Data Availability Statement

The original contributions presented in the study are included in the article/**Supplementary Material**. Further inquiries can be directed to the corresponding author.

## Author Contributions

Each author contributed significantly to concept and development of the present paper. LYY and MZ designed the research process. WD and MZ searched the database for corresponding articles and extracted useful information from the articles above. YXS and YTS used statistical software for analysis. XL, KJ, and JS drafted the meta-analysis. All authors had read and approved the manuscript and ensured that this was the case.

## Conflict of Interest

The authors declare that the research was conducted in the absence of any commercial or financial relationships that could be construed as a potential conflict of interest.

## Publisher’s Note

All claims expressed in this article are solely those of the authors and do not necessarily represent those of their affiliated organizations, or those of the publisher, the editors and the reviewers. Any product that may be evaluated in this article, or claim that may be made by its manufacturer, is not guaranteed or endorsed by the publisher.

## Supplementary Material

Supplementary Table 1 Characteristics of all studies included in the meta-analysis.

Supplementary Table 2 Quality assessment of included studies in this meta-analysis.

## Abbreviations

MIS, minimally invasive surgery; ARH, Abdominal radical hysterectomy; OS, overall survival; DFS, disease-free survival; HR, hazard ratio; CI, confidence interval; FIGO, International Federation of Gynecology and Obstetrics; RCT, randomized controlled trial.

## References

[1] Sung H Ferlay J Siegel RL Laversanne M Soerjomataram I Jemal A . Global Cancer Statistics 2020: GLOBOCAN Estimates of Incidence and Mortality Worldwide for 36 Cancers in 185 Countries. CA: A Cancer J Clin (2021) 71(3):209–49. doi: 10.3322/caac.21660 33538338

[2] Koh WJ Abu-Rustum NR Bean S Bradley K Campos SM Cho KR . Cervical Cancer, Version 3.2019, NCCN Clinical Practice Guidelines in Oncology. J Natl Compr Canc Netw (2019) 17(1):64–84. doi: 10.6004/jnccn.2019.0001 30659131

[3] Cibula D Pötter R Planchamp F Avall-Lundqvist E Fischerova D Haie Meder C . The European Society of Gynaecological Oncology/European Society for Radiotherapy and Oncology/European Society of Pathology Guidelines for the Management of Patients With Cervical Cancer. Radiother Oncol (2018) 127(3):404–16. doi: 10.1016/j.radonc.2018.03.003 29728273

[4] Nezhat CR Burrell MO Nezhat FR Benigno BB Welander CE . Laparoscopic Radical Hysterectomy With Paraaortic and Pelvic Node Dissection. Am J Obstetrics Gynecology (1992) 166(3):864–5. doi: 10.1016/0002-9378(92)91351-A 1532291

[5] Abu-Rustum NR Hoskins WJ . Radical Abdominal Hysterectomy. Surg Clin North Am (2001) 81(4):815–28. doi: 10.1016/S0039-6109(05)70167-5 11551127

[6] Spirtos NM Eisenkop SM Schlaerth JB Ballon SC . Laparoscopic Radical Hysterectomy (Type III) With Aortic and Pelvic Lymphadenectomy in Patients With Stage I Cervical Cancer: Surgical Morbidity and Intermediate Follow-Up. Am J Obstet Gynecol (2002) 187(2):340–8. doi: 10.1067/mob.2002.123035 12193922

[7] Stewart KI Fader AN . New Developments in Minimally Invasive Gynecologic Oncology Surgery. Clin Obstet Gynecol (2017) 60(2):330–48. doi: 10.1097/GRF.0000000000000286 PMC5576030 28406810

[8] Koh WU Greer BE Abu-Rustum NR Apte SM Campos SM Cho KR . Cervical Cancer, Version 2.2015. J Natl Compr Cancer Network JNCCN (2015) 13(4):395–404. doi: 10.6004/jnccn.2015.0055 25870376

[9] Clayton RD . Hysterectomy. Best Pract Res Clin Obstet Gynaecol (2006) 20(1):73–87. doi: 10.1016/j.bpobgyn.2005.09.007 16275095

[10] Wenzel HHB Smolders RGV Beltman JJ Lambrechts S Trum HW Yigit R . Survival of Patients With Early-Stage Cervical Cancer After Abdominal or Laparoscopic Radical Hysterectomy: A Nationwide Cohort Study and Literature Review. Eur J Cancer (2020) 133:14–21. doi: 10.1016/j.ejca.2020.04.006 32422504

[11] Yuan Z Cao D Yang J Yu M Shen K Yang J . Laparoscopic vs. Open Abdominal Radical Hysterectomy for Cervical Cancer: A Single-Institution, Propensity Score Matching Study in China. Front Oncol (2019) 9:1107. doi: 10.3389/fonc.2019.01107 31737563 PMC6833183

[12] Ramirez PT Frumovitz M Pareja R Lopez A Vieira M Ribeiro R . Minimally Invasive Versus Abdominal Radical Hysterectomy for Cervical Cancer. N Engl J Med (2018) 379(20):1895–904. doi: 10.1056/NEJMoa1806395 30380365

[13] Committee FGO . FIGO Statement on Minimally Invasive Surgery in Cervical Cancer. Int J Gynaecol Obstet (2020) 149(3):264. doi: 10.1002/ijgo.13141 32301115

[14] Wang W Li L Wu M Ma S Tan X Zhong S . Laparoscopic vs. Abdominal Radical Hysterectomy for Locally Advanced Cervical Cancer. Front Oncol (2019) 9:1331. doi: 10.3389/fonc.2019.01331 31828044 PMC6890871

[15] Uppal S Gehrig PA Peng K Bixel KL Matsuo K Vetter MH . Recurrence Rates in Patients With Cervical Cancer Treated With Abdominal Versus Minimally Invasive Radical Hysterectomy: A Multi-Institutional Retrospective Review Study. J Clin Oncol Off J Am Soc Clin Oncol (2020) 38(10):1030–40. doi: 10.1200/JCO.19.03012 32031867

[16] Kim SI Lee M Lee S Suh DH Kim HS Kim K . Impact of Laparoscopic Radical Hysterectomy on Survival Outcome in Patients With FIGO Stage IB Cervical Cancer: A Matching Study of Two Institutional Hospitals in Korea. Gynecol Oncol (2019) 155(1):75–82. doi: 10.1016/j.ygyno.2019.07.019 31383569

[17] Paik ES Lim MC Kim MH Kim YH Song ES Seong SJ . Comparison of Laparoscopic and Abdominal Radical Hysterectomy in Early Stage Cervical Cancer Patients Without Adjuvant Treatment: Ancillary Analysis of a Korean Gynecologic Oncology Group Study (KGOG 1028). Gynecol Oncol (2019) 154(3):547–53. doi: 10.1016/j.ygyno.2019.06.023 31272738

[18] Gu L Huang X Li S Mao D Shen Z Khadaroo PA . A Meta-Analysis of the Medium- and Long-Term Effects of Laparoscopic Sleeve Gastrectomy and Laparoscopic Roux-En-Y Gastric Bypass. BMC Surg (2020) 20(1):30. doi: 10.1186/s12893-020-00695-x 32050953 PMC7014764

[19] Gu L Du N Jin Q Li S Xie L Mo J . Magnitude of Benefit of the Addition of Poly ADP-Ribose Polymerase (PARP) Inhibitors to Therapy for Malignant Tumor: A Meta-Analysis. Crit Rev Oncol Hematol (2020) 147:102888. doi: 10.1016/j.critrevonc.2020.102888 32018126

[20] Gu L Chen M Guo D Zhu H Zhang W Pan J . PD-L1 and Gastric Cancer Prognosis: A Systematic Review and Meta-Analysis. PloS One (2017) 12(8):e0182692. doi: 10.1371/journal.pone.0182692 28796808 PMC5552131

[21] Li LY Wen LY Park SH Nam EJ Lee JY Kim S . Impact of the Learning Curve on the Survival of Abdominal or Minimally Invasive Radical Hysterectomy for Early-Stage Cervical Cancer. Cancer Res Treat (2021) 53(1):243–51. doi: 10.4143/crt.2020.063 PMC7811999 33070554

[22] Kim S Min KJ Lee S Hong JH Song JY Lee JK . Learning Curve Could Affect Oncologic Outcome of Minimally Invasive Radical Hysterectomy for Cervical Cancer. Asian J Surg (2021) 44(1):174–80. doi: 10.1016/j.asjsur.2020.05.006 32467009

[23] Kim SI Lee J Hong J Lee SJ Park DC Yoon JH . Comparison of Abdominal and Minimally Invasive Radical Hysterectomy in Patients With Early Stage Cervical Cancer. Int J Med Sci (2021) 18(5):1312–7. doi: 10.7150/ijms.55017 PMC7847619 33526992

[24] Zaccarini F Santy A Dabi Y Lavoue V Carcopino X Bendifallah S . Comparison of Survival Outcomes Between Laparoscopic and Abdominal Radical Hysterectomy for Early-Stage Cervical Cancer: A French Multicentric Study. J Gynecol Obstet Hum Reprod (2021) 50(2):102046. doi: 10.1016/j.jogoh.2020.102046 33340751

[25] Chiva L Zanagnolo V Querleu D Martin-Calvo N Arévalo-Serrano J Căpîlna ME . SUCCOR Study: An International European Cohort Observational Study Comparing Minimally Invasive Surgery Versus Open Abdominal Radical Hysterectomy in Patients With Stage IB1 Cervical Cancer. Int J Gynecol Cancer (2020) 30(9):1269–77. doi: 10.1136/ijgc-2020-001506 32788262

[26] Levine MD Brown J Crane EK Tait DL Naumann RW . Outcomes of Minimally Invasive Versus Open Radical Hysterectomy for Early Stage Cervical Cancer Incorporating 2018 FIGO Staging. J Minim Invasive Gynecol (2021) 28(4):824–8. doi: 10.1016/j.jmig.2020.07.021 32730990

[27] Gil-Moreno A Carbonell-Socias M Salicrú S Centeno-Mediavilla C Franco-Camps S Colas E . Radical Hysterectomy: Efficacy and Safety in the Dawn of Minimally Invasive Techniques. J Minim Invasive Gynecol (2019) 26(3):492–500. doi: 10.1016/j.jmig.2018.06.007 29908339

[28] Cusimano MC Baxter NN Gien LT Moineddin R Liu N Dossa F . Impact of Surgical Approach on Oncologic Outcomes in Women Undergoing Radical Hysterectomy for Cervical Cancer. Am J Obstet Gynecol (2019) 221(6):619 e1–619 e24. doi: 10.1016/j.ajog.2019.07.009 31288006

[29] Campos L Limberger LF Stein AT Caldas JM . Survival After Laparoscopic Versus Abdominal Radical Hysterectomy in Early Cervical Cancer: A Randomized Controlled Trial. Asian Pacific J Cancer Prev (2021) 22(1):93–7. doi: 10.31557/APJCP.2021.22.1.93 PMC8184196 33507684

[30] Rodriguez J Rauh-Hain JA Saenz J Isla DO Rendon Pereira GJ Odetto D . Oncological Outcomes of Laparoscopic Radical Hysterectomy Versus Radical Abdominal Hysterectomy in Patients With Early-Stage Cervical Cancer: A Multicenter Analysis. Int J Gynecol Cancer (2021) 31(4):504–11. doi: 10.1136/ijgc-2020-002086 33504547

[31] Li P Chen L Ni Y Liu J Li D Guo J . Comparison Between Laparoscopic and Abdominal Radical Hysterectomy for Stage IB1 and Tumor Size <2 Cm Cervical Cancer With Visible or Invisible Tumors: A Multicentre Retrospective Study. J Gynecol Oncol (2021) 32(2):e17. doi: 10.3802/jgo.2021.32.e17 33470062 PMC7930457

[32] Dai D Huang H Feng Y Wan T Liu Z Tong C . Minimally Invasive Surgery vs Laparotomy for Early Stage Cervical Cancer: A Propensity Score-Matched Cohort Study. Cancer Med (2020) 9(24):9236–45. doi: 10.1002/cam4.3527 PMC7774733 33236825

[33] Abel MK Chan JK Chow S Darcy K Tian C Kapp DS . Trends and Survival Outcomes of Robotic, Laparoscopic, and Open Surgery for Stage II Uterine Cancer. Int J Gynecol Cancer (2020) 30(9):1347–55. doi: 10.1136/ijgc-2020-001646 32753561

[34] Kwon BS Roh HJ Lee S Yang J Song YJ Lee SH . Comparison of Long-Term Survival of Total Abdominal Radical Hysterectomy and Laparoscopy-Assisted Radical Vaginal Hysterectomy in Patients With Early Cervical Cancer: Korean Multicenter, Retrospective Analysis. Gynecol Oncol (2020) 159(3):642–8. doi: 10.1016/j.ygyno.2020.09.035 33041070

[35] Qin M Siyi L Huang HF Li Y Gu Y Wang W . A Comparison of Laparoscopies and Laparotomies for Radical Hysterectomy in Stage IA1-IB1 Cervical Cancer Patients: A Single Team With 18 Years of Experience. Front Oncol (2020) 10:1738. doi: 10.3389/fonc.2020.01738 32984056 PMC7485394

[36] Hu TWY Huang Y Li N Nie D Li Z . Comparison of Laparoscopic Versus Open Radical Hysterectomy in Patients With Early-Stage Cervical Cancer: A Multicenter Study in China. Int J Gynecol Cancer (2020) 30(8):1143–50. doi: 10.1136/ijgc-2020-001340 32571892

[37] Chen X Zhao N Ye P Chen J Nan X Zhao H . Comparison of Laparoscopic and Open Radical Hysterectomy in Cervical Cancer Patients With Tumor Size </=2 Cm. Int J Gynecol Cancer (2020) 30(5):564–71. doi: 10.1136/ijgc-2019-000994 32276941

[38] Pedone Anchora L Turco LC Bizzarri N Capozzi VA Lombisani A Chiantera V . How to Select Early-Stage Cervical Cancer Patients Still Suitable for Laparoscopic Radical Hysterectomy: A Propensity-Matched Study. Ann Surg Oncol (2020) 27(6):1947–55. doi: 10.1245/s10434-019-08162-5 31898100

[39] Liu Y Li L Wu M Ma S Tan X Zhong S . The Impact of the Surgical Routes and Learning Curve of Radical Hysterectomy on the Survival Outcomes in Stage IB Cervical Cancer: A Retrospective Cohort Study. Int J Surg (2019) 68:72–7. doi: 10.1016/j.ijsu.2019.06.009 31220631

[40] Lim TYK Lin KKM Wong WL Aggarwal IM Yam PKL . Surgical and Oncological Outcome of Total Laparoscopic Radical Hysterectomy Versus Radical Abdominal Hysterectomy in Early Cervical Cancer in Singapore. Gynecol Minim Invasive Ther (2019) 8(2):53–8. doi: 10.4103/GMIT.GMIT_43_18 PMC6515754 31143623

[41] Guo J Yang L Cai J Xu L Min J Shen Y . Laparoscopic Procedure Compared With Open Radical Hysterectomy With Pelvic Lymphadenectomy in Early Cervical Cancer: A Retrospective Study. Onco Targets Ther (2018) 11:5903–8. doi: 10.2147/OTT.S156064 PMC6151097 30271174

[42] Corrado G Vizza E Legge F Pedone Anchora L Sperduti I Fagotti A . Comparison of Different Surgical Approaches for Stage IB1 Cervical Cancer Patients: A Multi-Institution Study and a Review of the Literature. Int J Gynecol Cancer (2018) 28(5):1020–8. doi: 10.1097/IGC.0000000000001254 29727351

[43] Brandt B Sioulas V Basaran D Kuhn T LaVigne K Gardner GJ . Minimally Invasive Surgery Versus Laparotomy for Radical Hysterectomy in the Management of Early-Stage Cervical Cancer: Survival Outcomes. Gynecol Oncol (2020) 156(3):591–7. doi: 10.1016/j.ygyno.2019.12.038 PMC7056548 31918996

[44] Park JY Kim D Suh DS Kim JH Kim YM Kim YT . The Role of Laparoscopic Radical Hysterectomy in Early-Stage Adenocarcinoma of the Uterine Cervix. Ann Surg Oncol (2016) 23(Suppl 5):825–33. doi: 10.1245/s10434-016-5489-4 27503491

[45] Mendivil AA Rettenmaier MA Abaid LN Brown JV 3rd Micha JP Lopez KL . Survival Rate Comparisons Amongst Cervical Cancer Patients Treated With an Open, Robotic-Assisted or Laparoscopic Radical Hysterectomy: A Five Year Experience. Surg Oncol (2016) 25(1):66–71. doi: 10.1016/j.suronc.2015.09.004 26409687

[46] Ditto A Martinelli F Bogani G Gasparri ML Di Donato V Zanaboni F . Implementation of Laparoscopic Approach for Type B Radical Hysterectomy: A Comparison With Open Surgical Operations. Eur J Surg Oncol (2015) 41(1):34–9. doi: 10.1016/j.ejso.2014.10.058 25468458

[47] Toptas T Simsek T . Total Laparoscopic Versus Open Radical Hysterectomy in Stage IA2-IB1 Cervical Cancer: Disease Recurrence and Survival Comparison. J Laparoendosc Adv Surg Tech A (2014) 24(6):373–8. doi: 10.1089/lap.2013.0514 24742012

[48] Kong TW Chang SJ Lee J Paek J Ryu HS . Comparison of Laparoscopic Versus Abdominal Radical Hysterectomy for FIGO Stage IB and IIA Cervical Cancer With Tumor Diameter of 3 Cm or Greater. Int J Gynecol Cancer (2014) 24(2):280–8. doi: 10.1097/IGC.0000000000000052 24407571

[49] van de Lande J von Mensdorff-Pouilly S Lettinga RG Piek JM Verheijen RH . Open Versus Laparoscopic Pelvic Lymph Node Dissection in Early Stage Cervical Cancer: No Difference in Surgical or Disease Outcome. Int J Gynecol Cancer (2012) 22(1):107–14. doi: 10.1097/IGC.0b013e31822c273d 21857347

[50] Choi CH Lee JW Lee YY Kim HJ Song T Kim MK . Comparison of Laparoscopic-Assisted Radical Vaginal Hysterectomy and Laparoscopic Radical Hysterectomy in the Treatment of Cervical Cancer. Ann Surg Oncol (2012) 19(12):3839–48. doi: 10.1245/s10434-012-2406-3 22644508

[51] Lee EJ Kang H Kim DH . A Comparative Study of Laparoscopic Radical Hysterectomy With Radical Abdominal Hysterectomy for Early-Stage Cervical Cancer: A Long-Term Follow-Up Study. Eur J Obstet Gynecol Reprod Biol (2011) 156(1):83–6. doi: 10.1016/j.ejogrb.2010.12.016 21269754

[52] Sobiczewski P Bidzinski M Derlatka P Panek G Danska-Bidzinska A Gmyrek L . Early Cervical Cancer Managed by Laparoscopy and Conventional Surgery: Comparison of Treatment Results. Int J Gynecol Cancer (2009) 19(8):1390–5. doi: 10.1111/IGC.0b013e3181ba5e88 20009895

[53] Malzoni M Tinelli R Cosentino F Fusco A Malzoni C . Total Laparoscopic Radical Hysterectomy Versus Abdominal Radical Hysterectomy With Lymphadenectomy in Patients With Early Cervical Cancer: Our Experience. Ann Surg Oncol (2009) 16(5):1316–23. doi: 10.1245/s10434-009-0342-7 19224286

[54] Jackson KS Das N Naik R Lopes AD Godfrey KA Hatem MH . Laparoscopically Assisted Radical Vaginal Hysterectomy vs. Radical Abdominal Hysterectomy for Cervical Cancer: A Match Controlled Study. Gynecol Oncol (2004) 95(3):655–61. doi: 10.1016/j.ygyno.2004.07.055 15581978

[55] Chen B Ji M Li P Liu P Zou W Zhao Z . Comparison Between Robot-Assisted Radical Hysterectomy and Abdominal Radical Hysterectomy for Cervical Cancer: A Multicentre Retrospective Study. Gynecol Oncol (2020) 157(2):429–36. doi: 10.1016/j.ygyno.2020.02.019 32067814

[56] Yang J Mead-Harvey C Polen-De C Magtibay P Butler K Cliby W . Survival Outcomes in Patients With Cervical Cancer Treated With Open Versus Robotic Radical Hysterectomy: Our Surgical Pathology Interrogation. Gynecol Oncol (2020) 159(2):373–80. doi: 10.1016/j.ygyno.2020.08.031 32893029

[57] Doo DW Kirkland CT Griswold LH McGwin G Huh WK Leath CA 3rd . Comparative Outcomes Between Robotic and Abdominal Radical Hysterectomy for IB1 Cervical Cancer: Results From a Single High Volume Institution. Gynecol Oncol (2019) 153(2):242–7. doi: 10.1016/j.ygyno.2019.03.001 PMC6818647 30850169

[58] Alfonzo E Wallin E Ekdahl L Staf C Rådestad AF Reynisson P . No Survival Difference Between Robotic and Open Radical Hysterectomy for Women With Early-Stage Cervical Cancer: Results From a Nationwide Population-Based Cohort Study. Eur J Cancer (2019) 116:169–77. doi: 10.1016/j.ejca.2019.05.016 31200323

[59] Shah CA Beck T Liao JB Giannakopoulos NV Veljovich D Paley P . Surgical and Oncologic Outcomes After Robotic Radical Hysterectomy as Compared to Open Radical Hysterectomy in the Treatment of Early Cervical Cancer. J Gynecol Oncol (2017) 28(6):e82. doi: 10.3802/jgo.2017.28.e82 29027400 PMC5641532

[60] Sert BM Boggess JF Ahmad S Jackson AL Stavitzski NM Dahl AA . Robot-Assisted Versus Open Radical Hysterectomy: A Multi-Institutional Experience for Early-Stage Cervical Cancer. Eur J Surg Oncol (2016) 42(4):513–22. doi: 10.1016/j.ejso.2015.12.014 26843445

[61] Jensen PT Schnack TH Frøding LP Bjørn SF Lajer H Markauskas A . Survival After a Nationwide Adoption of Robotic Minimally Invasive Surgery for Early-Stage Cervical Cancer – A Population-Based Study. Eur J Cancer (2020) 128:47–56. doi: 10.1016/j.ejca.2019.12.020 32109850

[62] Naumann RW . Minimally Invasive Radical Hysterectomy Has Many Benefits Compared With Open Radical Hysterectomy: Will the LACC Trial Cause the Premature Demise of This Procedure? J Minim Invasive Gynecol (2019) 26(3):379–80. doi: 10.1016/j.jmig.2019.01.002 30639317

[63] Sert BM Kristensen GB Kleppe A Dørum A . Long-Term Oncological Outcomes and Recurrence Patterns in Early-Stage Cervical Cancer Treated With Minimally Invasive Versus Abdominal Radical Hysterectomy: The Norwegian Radium Hospital Experience. Gynecol Oncol (2021) 162(2):284–91. doi: 10.1016/j.ygyno.2021.05.028 34083029

[64] Chen C Fang Z Wang Q Li W Li P Wang L . Comparative Study on the Oncological Prognosis of Laparoscopy and Laparotomy for Stage IIA1 Cervical Squamous Cell Carcinoma. Eur J Surg Oncol (2021) 47(2):346–52. doi: 10.1016/j.ejso.2020.07.016 32859433

[65] Wagner AE Pappas L Ghia AJ Gaffney DK . Impact of Tumor Size on Survival in Cancer of the Cervix and Validation of Stage IIA1 and IIA2 Subdivisions. Gynecol Oncol (2013) 129(3):517–21. doi: 10.1016/j.ygyno.2013.03.008 23528928

[66] Armbrust R Chen F Richter R Muallem MZ Mustea A Holthaus B . Results of a German Wide Survey Towards Current Surgical Approach in Early Stage Cervical Cancer NOGGO MONITOR 11. Sci Rep (2021) 11(1):9774. doi: 10.1038/s41598-021-89071-0 33963213 PMC8105313

[67] Fagotti A Pedone Anchora L Conte C Chiantera V Vizza E Tortorella L . Beyond Sentinel Node Algorithm. Toward a More Tailored Surgery for Cervical Cancer Patients. Cancer Med (2016) 5(8):1725–30. doi: 10.1002/cam4.722 PMC4971900 27230108

[68] Gulseren V Kocaer M Gungorduk O Ozdemir IA Gokcu M Mart EM . Preoperative Predictors of Pelvic and Para-Aortic Lymph Node Metastases in Cervical Cancer. J Cancer Res Ther (2019) 15(6):1231–4. doi: 10.4103/jcrt.JCRT_467_17 31898653

[69] Casarin J Buda A Bogani G Fanfani F Papadia A Ceccaroni M . Predictors of Recurrence Following Laparoscopic Radical Hysterectomy for Early-Stage Cervical Cancer: A Multi-Institutional Study. Gynecol Oncol (2020) 159(1):164–70. doi: 10.1016/j.ygyno.2020.06.508 32665147

[70] Eoh KJ Lee JY Nam EJ Kim S Kim SW Kim YT . The Institutional Learning Curve is Associated With Survival Outcomes of Robotic Radical Hysterectomy for Early-Stage Cervical Cancer-a Retrospective Study. BMC Cancer (2020) 20(1):152. doi: 10.1186/s12885-020-6660-7 32093687 PMC7041237

[71] Wang Y Li B Ren F Song Z Ouyang L Liu K . Survival After Minimally Invasive vs. Open Radical Hysterectomy for Cervical Cancer: A Meta-Analysis. Front Oncol (2020) 10:1236. doi: 10.3389/fonc.2020.01236 32903313 PMC7396529

[72] Nica A Kim SR Gien LT Covens A Bernardini MQ Bouchard-Fortier G . Survival After Minimally Invasive Surgery in Early Cervical Cancer: Is the Intra-Uterine Manipulator to Blame? Int J Gynecol Cancer (2020) 30(12):1864–70. doi: 10.1136/ijgc-2020-001816 33037109

[73] Kim JH Kim K Park SJ Lee JY Kim K Lim MC . Comparative Effectiveness of Abdominal Versus Laparoscopic Radical Hysterectomy for Cervical Cancer in the Postdissemination Era. Cancer Res Treat (2019) 51(2):788–96. doi: 10.4143/crt.2018.120 PMC6473278 30205416

[74] Schermerhorn SMV Christman MS Rocco NR Abdul-Muhsin H L'Esperance JO Castle EP . Learning Curve for Robotic-Assisted Laparoscopic Retroperitoneal Lymph Node Dissection. J Endourol (2021) 35:1483–9. doi: 10.1089/end.2020.0549 33559522

[75] Lin F Pan L Li L Li D Mo L . Effects of a Simulated CO2 Pneumoperitoneum Environment on the Proliferation, Apoptosis, and Metastasis of Cervical Cancer Cells In Vitro. Med Sci Monit (2014) 20:2497–503. doi: 10.12659/MSM.891179 PMC4260668 25436974

[76] Kong TW Chang SJ Piao X Paek J Lee Y Lee EJ . Patterns of Recurrence and Survival After Abdominal Versus Laparoscopic/Robotic Radical Hysterectomy in Patients With Early Cervical Cancer. J Obstet Gynaecol Res (2016) 42(1):77–86. doi: 10.1111/jog.12840 26554751

