# Supplementary material for: Comparison of Minimally Invasive Versus Abdominal Radical Hysterectomy for Early-Stage Cervical Cancer: An Updated Meta-Analysis
Source: Front Oncol. 2022 Jan 24;11:762921. doi: 10.3389/fonc.2021.762921 (PMC8818747; doi:10.3389/fonc.2021.762921)
Supplement: Supplementary Table 1 — Characteristics of all studies included in the meta-analysis. [file DataSheet_1.docx]

**Supplementary Table 1-1 Characteristics of all the studies included in the meta-analysis.**

| Author | Year | Time to recruit patients | Laparoscopic type | Age, (year) median | | Median follow-up (month) | | LNM(positive,%) | | LVSI(positive,%) | | Postoperative adjuvant therapy (yes,%) | |
| --- | --- | --- | --- | --- | --- | --- | --- | --- | --- | --- | --- | --- | --- |
|  |  |  |  | MIS | Control | MIS | Control | MIS | Control | MIS | Control | MIS | Control |
| Li | 2021 | 2002-2018 | TLRH | 48 | 47 | 121.2 | 50.4 | 6.4 | 15.7 | 34.4 | 66.1 | 25.5 | 41.1 |
| Kim | 2021 | 2006.5-2016.12 | TLRH | NA | NA | NA | NA | 4.5 | 4.5 | 19.4 | 22.7 | NA | NA |
| Kim | 2021 | 2010.1-2019.12 | TLRH | 52 | 49 | 45.5 | 37.8 | NA | NA | 17.3 | 15.8 | 40.0 | 68.4 |
| Zaccarini | 2021 | 1996.1-2017.12 | TLRH | 48 | 51 | 39.6 | 57.4 | 8.3 | 15.6 | 24.2 | 29.3 | 47.1 | 53.7 |
| Chiva | 2020 | 2013.1-2014.12 | TLRH | 48 | 49 | 56.0 | 60.0 | 12.0 | 16.4 | 43.3 | 53.5 | 51.6 | 52.7 |
| Levine | 2020 | 2007-2018 | TLRH | 42 | 43 | 17.0 | 40.0 | NA | NA | 34.2 | 38.6 | 11.1 | 60.0 |
| Uppal | 2020 | 2010.1-2017.12 | TLRH | NA | NA | 30.8 | 44.5 | 12.1 | 10.6 | 38.4 | 36.1 | 30.9 | 36.5 |
| Gil-Moreno | 2019 | 1999.5-2016.6 | TLRH | NA | NA | 112.4 | 112.4 | 10.7 | 15.8 | NA | NA | 32.1 | 57.9 |
| Cusimano | 2019 | 2006-2017 | TLRH | 45 | 47 | 63.6 | 80.4 | NA | NA | NA | NA | 23.9 | 32.0 |
| Ramirez | 2018 | 2008-2017 | TLRH | 46.1 | 46 | 30 | 30 | 12.4 | 13.1 | 24.1 | 28.7 | 28.8 | 27.6 |
| Campos | 2021 | 1999-2004 | TLRH | NA | NA | 60.0 | 60.0 | NA | NA | NA | NA | 100 | 100 |
| Rodriguez | 2021 | 2006.1-2017.12 | TLRH | NA | NA | 52.1 | 52.6 | NA | NA | NA | NA | 29.2 | 30.7 |
| Li | 2021 | 2004.1-2016.12 | TLRH | 47 | 46 | 42.0 | 48.0 | 9.1 | 8.4 | 14.5 | 13.6 | 42.3 | 40.4 |
| Dai | 2020 | 2012.2-2015.12 | TLRH | 45 | 47 | 68.7 | 68.7 | NA | NA | 28.6 | 23.0 | 26.8 | 26.8 |
| Abel^#^ | 2020 | 2010-2015 | TLRH | 64 | 63 | NA | NA | NA | NA | NA | NA | 70.5 | 70.0 |
| Kwon | 2020 | 2008.1-2017.12 | Mixed | 50 | 49 | 80.4 | 82.2 | 7.1 | 11.6 | 33.3 | 36.0 | 33.7 | 39.9 |
| Qin | 2020 | 2000-2018 | TLRH | 44 | 43 | 59.0 | 59.0 | NA | NA | NA | NA | 37.2 | 44.0 |
| Hu | 2020 | 2013.1-2016.12 | TLRH | 45 | 45 | 60.0 | 60.0 | 15.3 | 13.3 | 31.5 | 29.6 | 59.9 | 58.4 |
| Chen | 2020 | 2010.1-2018.12 | TLRH | 49 | 52 | 51.8 | 49.5 | 0.8 | 5.6 | 13.2 | 16.3 | 23.1 | 31.6 |
| Wenzel | 2020 | 2010-2017 | TLRH | 44 | 46 | NA | NA | 8.0 | 18.0 | 41.0 | 47.0 | 15.0 | 28.0 |
| Pedone Anchora | 2020 | 2012.6-2016.12 | TLRH | 46 | 46 | 42.0 | 49.0 | 8.3 | 19.8 | 33.0 | 41.0 | 34.0 | 32.7 |
| Wang | 2019 | 2001.2-2015.11 | TLRH | 45 | 46 | 41.3 | 41.3 | 19.8 | 17.3 | 40.1 | 39.7 | 95.4 | 92.7 |
| Yuan | 2019 | 2012.1-2014.12 | TLRH | 44 | 45 | 59.0 | 69.0 | 11.1 | 10.1 | 23.2 | 34.3 | 21.2 | 22.2 |
| Kim | 2019 | 2000-2018 | TLRH | 50 | 49 | 112.5 | 34.5 | 12.2 | 12.6 | 37.4 | 38.3 | 35.6 | 44.6 |
| Paik | 2019 | 2000-2008 | TLRH | 46 | 46 | 69.1 | 63.9 | 0.0 | 0.0 | 18.5 | 19.6 | 0.0 | 0.0 |
| Liu | 2019 | 2001.2-2015.11 | TLRH | 43 | 43 | 49.0 | 49.0 | 15.5 | 11.1 | 62.7 | 45.2 | 52.4 | 55.6 |
| Lim | 2019 | 2009.11-2014.12 | TLRH | 47 | 49 | 29.2 | 35.8 | 13.7 | 14.1 | 33.3 | 41.2 | 43.1 | 55.3 |
| Guo | 2018 | 2008.5-2013.12 | TLRH | 44 | 41 | 39.0 | 39.0 | 12.9 | 14.4 | NA | NA | NA | NA |
| Corrado* | 2018 | 2001.1-2016.12 | TLRH | 45 | 50 | 41.7 | 82.1 | NA | NA | 35.5 | 36.6 | 37.5 | 37.6 |
| Wang | 2016 | 2002-2005 | TLRH | 45 | 44 | 68.3 | 68.3 | 12.8 | 12.8 | 16.3 | 16.3 | 33.3 | 53.2 |
| Park | 2016 | 1997-2013 | TLRH | 45 | 47 | 47.2 | 70.6 | 15.6 | 15.0 | 19.9 | 19.6 | 34.4 | 41.1 |
| Mendivil^$^ | 2016 | 2009.1-2013.12 | TLRH | 48 | 51 | 39.0 | 39.0 | 4.8 | 6.0 | NA | NA | 67.3 | 71.8 |
| Ditto | 2015 | 2002.2-2013.10 | TLRH | 46 | 46 | 31.0 | 48.7 | 5.0 | 10.0 | 38.0 | 50.0 | 22 | 23 |
| Toptas | 2014 | 2007-2010 | TLRH | 47 | 50 | 42.5 | 43.5 | 9.1 | 6.7 | 31.8 | 54.3 | 39.1 | 27.3 |
| Kong | 2014 | 2006.2-2013.3 | TLRH | 45 | 48 | 28.0 | 58.0 | 17.5 | 16.7 | 17.5 | 73.0 | 42.5 | 54.2 |
| van de Lande | 2012 | 2003-2005 | TLRH | 41 | 44 | 60.0 | 60.0 | NA | NA | NA | NA | 12.7 | 25.8 |
| Choi | 2012 | 2004.9-2010.12 | Mixed | 46 | 47 | 23.5 | 34.0 | 8.2 | 7.1 | 27.3 | 27.8 | NA | NA |
| Lee | 2011 | 1994.1-2001.12 | TLRH | 48 | 50 | 78.0 | 75.0 | 16.7 | 20.8 | NA | NA | 29.2 | 27.1 |
| Sobiczewski | 2009 | 2001.1-2004.12 | TLRH | 45 | 51 | 36.0 | 36.0 | 0.0 | 3.5 | 18.0 | 16.0 | 40.0 | 63.0 |
| Malzoni | 2009 | 1995.1-2007.5 | TLRH | 41 | 43 | 52.5 | 71.5 | NA | NA | NA | NA | NA | NA |
| Jackson | 2004 | 1996-2003 | LARVH | 46 | 46 | 52.0 | 49.0 | 2.0 | 2.0 | 32.0 | 24.0 | 14.0 | 0.0 |
| Abel^#^ | 2020 | 2010-2015 | TLRH | 64 | 63 | NA | NA | NA | NA | NA | NA | 73.7 | 70.0 |
| Chen | 2020 | 2004.1-2016.12 | TLRH | 48 | 48 | 24.0 | 48.0 | 14.7 | 15.8 | 22.3 | 24.3 | 69.7 | 70.4 |
| Yang | 2020 | 2000-2017 | TLRH | 47 | 46 | 53.0 | 130.0 | NA | NA | 26.3 | 27.3 | 16.4 | 32.0 |
| Doo | 2019 | 2010-2016 | TLRH | 44 | 41 | 25.4 | 25.4 | NA | NA | 26.0 | 35.0 | 18.0 | 34.0 |
| Alfonzo | 2019 | 2011.1-2017.12 | TLRH | 46 | 45 | 44.5 | 55.7 | 15.5 | 14.7 | 63.0 | 64.7 | 30.6 | 31.9 |
| Corrado* | 2018 | 2001.1-2016.12 | TLRH | 46 | 50 | 46.6 | 82.1 | NA | NA | 36.3 | 36.6 | 54.4 | 37.6 |
| Shah | 2017 | 2001-2012 | TLRH | 45 | 45 | 36.0 | 36.0 | 13.9 | 15.5 | 28.0 | 33.3 | 31.7 | 45.1 |
| Sert | 2016 | 2005-2011 | TLRH | 45 | 47 | 34.6 | 45.2 | 10.0 | 12.0 | 37.0 | 41.0 | 27.0 | 33.0 |
| Mendivil^$^ | 2016 | 2009.1-2013.12 | TLRH | 47 | 51 | 39.0 | 39.0 | 4.8 | 6.0 | NA | NA | 63.8 | 71.8 |
| Jensen | 2020 | 2005.1.1-2017.6.30 | LARVH | 43 | 43 | 42.4 | 113 | 7.6 | 11.0 | 31.1 | 36.4 | 27.9 | 31.9 |

MIS, Minimally invasive surgery; TLRH, Total laparoscopic radical hysterectomy; LARVH, Laparoscopic assisted radical vaginal hysterectomy; LNM, Lymph node metastasis; LVSI, Lymph-vascular space invasion; NA, Not available.

^#^Both were from the same study.

*Both were from the same study.

^$^Both were from the same study.
