# Supplementary material for: Comparison of Minimally Invasive Versus Abdominal Radical Hysterectomy for Early-Stage Cervical Cancer: An Updated Meta-Analysis
Source: Front Oncol. 2022 Jan 24;11:762921. doi: 10.3389/fonc.2021.762921 (PMC8818747; doi:10.3389/fonc.2021.762921)
Supplement: Supplementary Table 2 — Quality assessment of included studies in this meta-analysis. [file DataSheet_2.docx]

**Supplementary Table 1-2 Characteristics of all the studies included in the meta-analysis.**

| Author | Year | Tumor stage (FIGO, %) | | | | | | | | | | | | | | Differentiation (Grade, %) | | | | | | | |
| --- | --- | --- | --- | --- | --- | --- | --- | --- | --- | --- | --- | --- | --- | --- | --- | --- | --- | --- | --- | --- | --- | --- | --- |
|  |  | MIS | | | | | | | Control | | | | | | | MIS | | | | Control | | | |
|  |  | ⅠA1 | ⅠA2 | ⅠB1 | ⅠB2 | ⅠB3 | ⅡA | ⅡB | ⅠA1 | ⅠA2 | ⅠB1 | ⅠB2 | ⅠB3 | ⅡA | ⅡB | Grade 1 | Grade 2 | Grade 3 | Grade 4/ Unspecified | Grade 1 | Grade 2 | Grade 3 | Grade 4/ Unspecified |
| Li | 2021 | 9.6 | | 78.4 | 12.1 | | | 0 | 10 | | 67.9 | 22.1 | | | 0 | NA | NA | NA | NA | NA | NA | NA | NA |
| Kim | 2021 | NA | NA | NA | NA | NA | NA | NA | NA | NA | NA | NA | NA | NA | NA | NA | NA | NA | NA | NA | NA | NA | NA |
| Kim | 2021 | 0 | 0 | 62.7 | 23.6 | 9.1 | 4.6 | 0 | 0 | 0 | 26.3 | 50 | 15.8 | 7.9 | 0 | NA | NA | NA | NA | NA | NA | NA | NA |
| Zaccarini | 2021 | 9.4 | | 73.5 | | | 5.4 | 4 | 12.2 | | 78 | | | 0 | 7.3 | NA | NA | NA | NA | NA | NA | NA | NA |
| Chiva | 2020 | 0 | | 39.9 | 44.3 | 0 | 15.8 | | 0 | | 29.4 | 51.5 | 0 | 19.2 | | 14.4 | 44.7 | 24.7 | 6.2 | 14.7 | 49 | 28.6 | 7.7 |
| Levine | 2020 | 7.5 | | 80 | | 0 | 2.5 | 2.5 | 0 | | 65 | | 4 | 0 | 9 | NA | NA | NA | NA | NA | NA | NA | NA |
| Uppal | 2020 | 1.6 | 9.5 | 88.9 | 0 | 0 | 0 | 0 | 0.8 | 7.5 | 91.8 | 0 | 0 | 0 | 0 | 19.5 | 42.9 | 24.6 | 13 | 21.6 | 49.4 | 24.3 | 4.7 |
| Gil-Moreno | 2019 | NA | NA | 79.5 | 1.8 | NA | 6.3 | 0.9 | NA | NA | 82.9 | 1.3 | NA | 10.5 | 1.3 | 12.5 | 58.9 | 25.9 | 2.7 | 7.8 | 44.7 | 32.8 | 14.4 |
| Cusimano | 2019 | 28.5 | | | 54.1 | | 11.6 | | 28.1 | | | 57.3 | | 14.2 | | NA | NA | NA | NA | NA | NA | NA | NA |
| Ramirez | 2018 | 1.60 | 6.60 | 91.80 | NA | NA | NA | NA | 1.60 | 6.40 | 92.00 | NA | NA | NA | NA | NA | NA | NA | NA | NA | NA | NA | NA |
| Campos | 2021 | NA | NA | NA | NA | NA | NA | NA | NA | NA | NA | NA | NA | NA | NA | NA | NA | NA | NA | NA | NA | NA | NA |
| Rodriguez | 2021 | 1.3 | 6.5 | 92.2 | 0 | 0 | 0 | 0 | 2.2 | 5 | 92.8 | 0 | 0 | 0 | 0 | 20.5 | 56.4 | 14.8 | 8.3 | 10.2 | 62.7 | 14.9 | 12.2 |
| Li | 2021 | NA | NA | NA | NA | NA | NA | NA | NA | NA | NA | NA | NA | NA | NA | NA | NA | NA | NA | NA | NA | NA | NA |
| Dai | 2020 | 0 | 0 | 23.9 | 61 | 15 | 0 | 0 | 0 | 0 | 23.9 | 61 | 15 | 0 | 0 | 7.5 | 45.1 | 47.4 | 0 | 4.7 | 44.1 | 51.2 | 0 |
| Abel# | 2020 | NA | NA | NA | NA | NA | NA | NA | NA | NA | NA | NA | NA | NA | NA | 17.8 | 31.5 | 38 | 12.7 | 6.9 | 26 | 48 | 11.7 |
| Kwon | 2020 | 0 | 6.7 | 43.7 | 49.6 | 0 | 0 | 0 | 5 | 41.9 | 53.1 | 0 | 0 | 0 | 0 | NA | NA | NA | NA | NA | NA | NA | NA |
| Qin | 2020 | 1.7 | 8.1 | 90.2 | 0 | 0 | 0 | 0 | 1.2 | 3.5 | 95.3 | 0 | 0 | 0 | 0 | 56.4 | 25 | 18.6 | 0 | 42.9 | 27.4 | 29.7 | 0 |
| Hu | 2020 | 0 | 11.6 | 66.5 | 0 | 0 | 21.9 | 0 | 0 | 10.3 | 67.5 | 0 | 0 | 22.2 | 0 | NA | NA | NA | NA | NA | NA | NA | NA |
| Chen | 2020 | NA | NA | NA | NA | NA | NA | NA | NA | NA | NA | NA | NA | NA | NA | 19.4 | 41.9 | 27.9 | 10.9 | 17.4 | 30.6 | 36.2 | 15.8 |
| Wenzel | 2020 | 0 | 1 | 97 | 0 | 0 | 2 | 0 | 0 | 0 | 96 | 0 | 0 | 4 | 0 | 11 | 55 | 34 | 0 | 11 | 49 | 40 | 0 |
| Pedone Anchora | 2020 | 20.4 | | 68 | | | 5.3 | 6.3 | 9.7 | | 77 | | | 10.1 | 3.2 | 69.9 | | 30.1 | 0 | 53 | | 47 | 0 |
| Wang | 2019 | 0 | 0 | 0 | 68.2 | 0 | 23.5 | 8.3 | 0 | 0 | 0 | 54.2 | 0 | 35.1 | 10.6 | 12.9 | 50.2 | 36.9 | 0 | 3.9 | 43 | 53.1 | 0 |
| Yuan | 2019 | 73.7 | | | 26.3 | | | 0 | 72.7 | | | 27.3 | | | 0 | NA | NA | NA | NA | NA | NA | NA | NA |
| Kim | 2019 | 0 | 0 | 88.3 | 11.7 | 0 | 0 | 0 | 0 | 0 | 88.3 | 11.7 | 0 | 0 | 0 | NA | NA | NA | NA | NA | NA | NA | NA |
| Paik | 2019 | 0 | 0 | 97.5 | 0 | 0 | 2.5 | 0 | 0 | 0 | 97.5 | 0 | 0 | 2.5 | 0 | NA | NA | NA | NA | NA | NA | NA | NA |
| Liu | 2019 | 0 | 0 | 67.9 | 32.1 | 0 | 0 | 0 | 0 | 0 | 67.4 | 32.6 | 0 | 0 | 0 | 54.2 | 45.8 | | 0 | 41.5 | 58.5 | | 0 |
| Lim | 2019 | 13.7 | 3.9 | 76.5 | 5.9 | 0 | 0 | 0 | 8.2 | 0 | 72.9 | 16.5 | 0 | 2.4 | 0 | NA | NA | NA | NA | NA | NA | NA | NA |
| Guo | 2018 | 8.5 | | 80.3 | | | 11.2 | 0 | 8.6 | | 75.5 | | | 15.8 | 0 | 29.1 | 40.3 | 22.3 | 8.3 | 27.3 | 41.7 | 21.6 | 9.4 |
| Corrado* | 2018 | NA | NA | NA | NA | NA | NA | NA | NA | NA | NA | NA | NA | NA | NA | 8.5 | 56 | 25.5 | 0 | 5.9 | 46.5 | 47.5 | 0 |
| Wang | 2016 | 0 | 6.4 | 53.7 | 13.8 | 0 | 26.1 | 0 | 0 | 5.9 | 54.2 | 12.3 | 0 | 27.6 | 0 | 15.3 | 46.3 | 38.4 | 0 | 15.8 | 40.9 | 43.4 | 0 |
| Park | 2016 | 0 | 5.4 | 83.9 | 8.6 | 0 | 1.4 | 0 | 0 | 3.7 | 90.7 | 4.7 | 0 | 0.9 | 0 | 32.3 | 37.1 | 11.8 | 18.8 | 31.8 | 29 | 13.1 | 26.2 |
| Mendivil$ | 2016 | 0 | 8.2 | 36.7 | 30.6 | 0 | 18.4 | 6.1 | 0 | 5.1 | 38.5 | 33.3 | 0 | 17.9 | 5.1 | 22.4 | 34.7 | 42.9 | 0 | 20.5 | 43.6 | 35.9 | 0 |
| Ditto | 2015 | 0 | 22 | 88 | 0 | 0 | 0 | 0 | 0 | 17 | 83 | 0 | 0 | 0 | 0 | 57 | | 43 | 0 | 55 | | 45 | 0 |
| Toptas | 2014 | 0 | 40.9 | 59.1 | 0 | 0 | 0 | 0 | 0 | 15.3 | 84.7 | 0 | 0 | 0 | 0 | NA | NA | NA | NA | NA | NA | NA | NA |
| Kong | 2014 | 0 | 0 | 55 | 30 | 0 | 0 | 15 | 0 | 0 | 56.3 | 29.2 | 0 | 14.6 | 0 | 52.5 | 45 | 2.5 | 0 | 62.5 | 35.4 | 2.1 | 0 |
| van de Lande | 2012 | 0 | 0 | 89.5 | 10.5 | 0 | 0 | 0 | 0 | 0 | 88.2 | 11.8 | 0 | 0 | 0 | 9.2 | 46 | 44.7 | 0 | 3.2 | 37.6 | 59.1 | 0 |
| Choi | 2012 | 3.1 | 10.3 | 83 | 0 | 0 | 3.6 | 0 | 1 | 8.1 | 83.8 | 0 | 0 | 7.1 | 0 | NA | NA | NA | NA | NA | NA | NA | NA |
| Lee | 2011 | 0 | 20.8 | 54.2 | 8.3 | 0 | 16.7 | 0 | 0 | 20.8 | 54.2 | 8.3 | 0 | 16.7 | 0 | NA | NA | NA | NA | NA | NA | NA | NA |
| Sobiczewski | 2009 | 31 | | 68.2 | 0 | 0 | 0 | 0 | 13.7 | | 79 | 0 | 0 | 5.2 | 0 | 21 | 37 | 42 | 0 | 15 | 47 | 37 | 0 |
| Malzoni | 2009 | 7.7 | 32.3 | 60 | 0 | 0 | 0 | 0 | 4.8 | 17.7 | 77.4 | 0 | 0 | 0 | 0 | 61.5 | 21.5 | 17 | 0 | 64.5 | 27.4 | 8.1 | 0 |
| Jackson | 2004 | 0 | 4 | 94 | 2 | 0 | 0 | 0 | 0 | 4 | 94 | 2 | 0 | 0 | 0 | NA | NA | NA | NA | NA | NA | NA | NA |
| Abel# | 2020 | NA | NA | NA | NA | NA | NA | NA | NA | NA | NA | NA | NA | NA | NA | 19.1 | 29.2 | 35.9 | 15.8 | 6.9 | 26 | 48 | 11.7 |
| Chen | 2020 | 1.3 | | 61.4 | 7.2 | 0 | 31 | 0 | 1 | | 59 | 8.9 | 0 | 30 | 0 | NA | NA | NA | NA | NA | NA | NA | NA |
| Yang | 2020 | 0 | 11.4 | 70 | 13.8 | 0 | 4.9 | 0 | 0 | 4.2 | 73.7 | 16.6 | 0 | 5.6 | 0 | 16.8 | 31.5 | 44.2 | 7.5 | 16 | 31.1 | 44.5 | 8.4 |
| Doo | 2019 | NA | NA | NA | NA | NA | NA | NA | NA | NA | NA | NA | NA | NA | NA | 16 | 69 | 10 | 4 | 4 | 66 | 16 | 14 |
| Alfonzo | 2019 | 3.4 | 7.3 | 89.2 | 0 | 0 | 0 | 0 | 3.4 | 8.6 | 87.9 | 0 | 0 | 0 | 0 | 15.9 | 38.4 | 31.9 | 13.8 | 15.5 | 36.2 | 32.3 | 15.9 |
| Corrado* | 2018 | NA | NA | NA | NA | NA | NA | NA | NA | NA | NA | NA | NA | NA | NA | 4.5 | 56.8 | 38.7 | 0 | 5.9 | 46.5 | 47.5 | 0 |
| Shah | 2017 | 5 | 15 | 63 | 4 | 0 | 0 | 0 | 7 | 1 | 63 | 11 | 0 | 0 | 0 | NA | NA | NA | NA | NA | NA | NA | NA |
| Sert | 2016 | 14 | | 80 | 6 | | | 0 | 10 | | 80 | 10 | | | 0 | NA | NA | NA | NA | NA | NA | NA | NA |
| Mendivil$ | 2016 | 0 | 62.1 | 32.8 | 29.3 | 0 | 20.7 | 8.6 | 0 | 5.1 | 38.5 | 33.3 | 0 | 17.9 | 5.1 | 19 | 41.4 | 39.7 | 0 | 20.5 | 43.6 | 35.9 | 0 |
| Jensen | 2020 | 0 | 2.4 | 97.6 | 0 | 0 | 0 | 0 | 0 | 1.9 | 98.1 | 0 | 0 | 0 | 0 | NA | NA | NA | NA | NA | NA | NA | NA |

MIS, Minimally invasive surgery; FIGO, International Federation of Gynecology and Obstetrics; NA, Not available.

^#^Both were from the same study.

*Both were from the same study.

^$^Both were from the same study.
