# Supplementary material for: Comparison of Minimally Invasive Versus Abdominal Radical Hysterectomy for Early-Stage Cervical Cancer: An Updated Meta-Analysis
Source: Front Oncol. 2022 Jan 24;11:762921. doi: 10.3389/fonc.2021.762921 (PMC8818747; doi:10.3389/fonc.2021.762921)
Supplement: Supplementary file 3 [file DataSheet_3.doc]

**Supplementary Table 2.** Quality assessment of studies included.

| Author, year,  Study (RCT) | Sequence  Generation | Allocation  Concealment | Blinding | Incomplete  outcome data | Selective  outcome reporting | Free of  other bias |
| --- | --- | --- | --- | --- | --- | --- |
| Ramirez, 2018 | low risk | high risk | unclear risk | low risk | low risk | low risk |
| Campos, 2021 | low risk | low risk | low risk | low risk | unclear risk | unclear risk |

| Author, year,  Study (Observational) | **Selection (Out of 4)** | | | | **Comparability**  **(Out of 2)** | **Outcomes(Out of 3)** | | | **Total**  **(Out of 9)** |
| --- | --- | --- | --- | --- | --- | --- | --- | --- | --- |
|  | Representativeness of exposed cohort | Selection of nonexposed cohort | Ascertainment  of exposure | Outcome not present at the start of the study |  | Assessment of outcomes | Length of follow-up | Adequacy of follow-up |  |
| Li, 2021 | 1 | 1 | 1 | 1 | 1 | 1 | 1 | 1 | 8 |
| Kim, 2021 | 1 | 0 | 1 | 1 | 2 | 1 | 1 | 0 | 7 |
| Kim, 2021 | 1 | 1 | 1 | 1 | 2 | 1 | 0 | 1 | 8 |
| Zaccarini, 2021 | 1 | 1 | 1 | 1 | 1 | 1 | 1 | 1 | 8 |
| Chiva, 2020 | 1 | 1 | 1 | 1 | 2 | 1 | 1 | 1 | 9 |
| Levine, 2020 | 1 | 1 | 1 | 1 | 1 | 1 | 0 | 1 | 7 |
| Uppal, 2020 | 1 | 1 | 1 | 1 | 2 | 1 | 0 | 1 | 8 |
| Gil-Moreno, 2019 | 1 | 1 | 1 | 1 | 1 | 1 | 0 | 1 | 7 |
| Cusimano, 2019 | 1 | 1 | 1 | 1 | 1 | 1 | 1 | 0 | 7 |
| Rodriguez, 2021 | 1 | 0 | 1 | 1 | 2 | 1 | 1 | 0 | 7 |
| Li, 2021 | 1 | 0 | 1 | 1 | 2 | 1 | 1 | 1 | 8 |
| Dai, 2020 | 1 | 1 | 1 | 1 | 2 | 1 | 1 | 1 | 9 |
| Abel, 2020 | 1 | 0 | 0 | 1 | 2 | 1 | 1 | 0 | 6 |
| Kwon, 2020 | 1 | 1 | 1 | 1 | 1 | 1 | 1 | 1 | 8 |
| Qin, 2020 | 1 | 1 | 1 | 1 | 2 | 1 | 1 | 1 | 9 |
| Hu, 2020 | 1 | 1 | 0 | 1 | 2 | 1 | 1 | 0 | 7 |
| Chen, 2020 | 1 | 1 | 1 | 1 | 2 | 1 | 1 | 0 | 8 |
| Wenzel, 2020 | 1 | 1 | 1 | 1 | 2 | 1 | 1 | 0 | 8 |
| Pedone Anchora, 2020 | 1 | 1 | 1 | 1 | 2 | 1 | 1 | 1 | 9 |
| Wang, 2019 | 1 | 1 | 1 | 1 | 1 | 1 | 1 | 1 | 8 |
| Yuan, 2019 | 1 | 1 | 1 | 1 | 0 | 1 | 1 | 1 | 7 |
| Kim, 2019 | 1 | 1 | 0 | 1 | 2 | 1 | 1 | 1 | 8 |
| Paik, 2019 | 1 | 1 | 1 | 1 | 1 | 1 | 1 | 1 | 8 |
| Liu, 2019 | 1 | 1 | 1 | 1 | 2 | 1 | 1 | 1 | 9 |
| Lim, 2019 | 1 | 1 | 1 | 1 | 1 | 1 | 0 | 1 | 7 |
| Guo, 2018 | 1 | 1 | 1 | 1 | 1 | 1 | 0 | 1 | 7 |
| Corrado, 2018 | 1 | 1 | 1 | 1 | 0 | 1 | 1 | 0 | 6 |
| Wang, 2016 | 1 | 1 | 1 | 1 | 2 | 1 | 1 | 1 | 9 |
| Park, 2016 | 1 | 1 | 1 | 1 | 2 | 1 | 1 | 1 | 9 |
| Mendivil, 2016 | 1 | 1 | 1 | 1 | 0 | 1 | 1 | 1 | 7 |
| Ditto, 2015 | 1 | 1 | 1 | 1 | 1 | 1 | 1 | 1 | 8 |
| Toptas, 2014 | 1 | 1 | 1 | 1 | 1 | 1 | 0 | 1 | 7 |
| Kong, 2014 | 1 | 1 | 1 | 1 | 0 | 1 | 0 | 1 | 6 |
| van de Lande, 2012 | 1 | 1 | 1 | 1 | 2 | 1 | 1 | 1 | 9 |
| Choi, 2012 | 1 | 1 | 1 | 1 | 0 | 1 | 1 | 1 | 7 |
| Lee, 2011 | 1 | 1 | 1 | 1 | 0 | 1 | 1 | 1 | 7 |
| Sobiczewski, 2009 | 1 | 1 | 1 | 1 | 1 | 1 | 0 | 0 | 6 |
| Malzoni, 2009 | 1 | 1 | 1 | 1 | 0 | 1 | 1 | 1 | 7 |
| Jackson, 2004 | 1 | 1 | 1 | 1 | 0 | 1 | 0 | 1 | 6 |
| Chen, 2020 | 1 | 1 | 1 | 1 | 2 | 1 | 0 | 1 | 8 |
| Yang, 2020 | 1 | 0 | 1 | 1 | 2 | 1 | 1 | 1 | 8 |
| Doo, 2019 | 1 | 1 | 1 | 1 | 2 | 1 | 0 | 1 | 8 |
| Alfonzo, 2019 | 1 | 1 | 1 | 1 | 2 | 1 | 1 | 1 | 9 |
| Shah, 2017 | 1 | 1 | 1 | 1 | 2 | 1 | 0 | 1 | 8 |
| Sert, 2016 | 1 | 1 | 1 | 1 | 2 | 1 | 0 | 1 | 8 |
| Jensen, 2020 | 1 | 1 | 1 | 1 | 1 | 1 | 1 | 1 | 8 |

The RCTs and observational studies were assessed by the Cochrane Collaboration’s tool and Newcastle-Ottawa Quality Assessment Scale, respectively.

Risk of bias was assessed as “low risk”, “high risk” or “unclear risk”.
